# Genomic signatures accompanying the dietary shift to phytophagy in polyphagan beetles

**DOI:** 10.1186/s13059-019-1704-5

**Published:** 2019-05-17

**Authors:** Mathieu Seppey, Panagiotis Ioannidis, Brent C. Emerson, Camille Pitteloud, Marc Robinson-Rechavi, Julien Roux, Hermes E. Escalona, Duane D. McKenna, Bernhard Misof, Seunggwan Shin, Xin Zhou, Robert M. Waterhouse, Nadir Alvarez

**Affiliations:** 1 Department of Ecology and Evolution, University of Lausanne, 1015 Lausanne, Switzerland; 2 Present address: Department of Genetic Medicine and Development, University of Geneva and Swiss Institute of Bioinformatics, 1211 Geneva, Switzerland; 3 Department of Genetic Medicine and Development, University of Geneva, 1211 Geneva, Switzerland; 4 Swiss Institute of Bioinformatics, 1015 Lausanne, Switzerland; 5 Present address: Institute of Molecular Biology and Biotechnology, Foundation for Research and Technology–Hellas, Heraklion, Greece; 6 Island Ecology and Evolution Research Group, Instituto de Productos Naturales y Agrobiología (IPNA-CSIC), San Cristóbal de la Laguna, Santa Cruz de Tenerife, Spain; 7 Present address: Department of Environmental Systems Science, ETHZ, 8092 Zurich, Switzerland; 8 Present address: Department of Biomedicine, University of Basel, 4031 Basel, Switzerland; 9 Center for Molecular Biodiversity Research (ZMB), Zoological Research Museum Alexander Koenig, 53113 Bonn, Germany; 10 Department of Biological Sciences, University of Memphis, Memphis, TN 38111 USA; 11 Department of Entomology, College of Plant Protection, China Agricultural University, Beijing, China; 12 Geneva Natural History Museum, 1208 Geneva, Switzerland

**Keywords:** Gene family evolution, Beetle-plant trophic interactions, Beetle diversification, Detoxification enzymes

## Abstract

**Background:**

The diversity and evolutionary success of beetles (Coleoptera) are proposed to be related to the diversity of plants on which they feed. Indeed, the largest beetle suborder, Polyphaga, mostly includes plant eaters among its approximately 315,000 species. In particular, plants defend themselves with a diversity of specialized toxic chemicals. These may impose selective pressures that drive genomic diversification and speciation in phytophagous beetles. However, evidence of changes in beetle gene repertoires driven by such interactions remains largely anecdotal and without explicit hypothesis testing.

**Results:**

We explore the genomic consequences of beetle-plant trophic interactions by performing comparative gene family analyses across 18 species representative of the two most species-rich beetle suborders. We contrast the gene contents of species from the mostly plant-eating suborder Polyphaga with those of the mainly predatory Adephaga. We find gene repertoire evolution to be more dynamic, with significantly more adaptive lineage-specific expansions, in the more speciose Polyphaga. Testing the specific hypothesis of adaptation to plant feeding, we identify families of enzymes putatively involved in beetle-plant interactions that underwent adaptive expansions in Polyphaga. There is notable support for the selection hypothesis on large gene families for glutathione S-transferase and carboxylesterase detoxification enzymes.

**Conclusions:**

Our explicit modeling of the evolution of gene repertoires across 18 species identifies putative adaptive lineage-specific gene family expansions that accompany the dietary shift towards plants in beetles. These genomic signatures support the popular hypothesis of a key role for interactions with plant chemical defenses, and for plant feeding in general, in driving beetle diversification.

**Electronic supplementary material:**

The online version of this article (10.1186/s13059-019-1704-5) contains supplementary material, which is available to authorized users.

## Background

Species richness among eukaryotes varies substantially, with some clades having only a few representatives and others comprising hundreds of thousands of extant species. In particular, the class Insecta outnumbers all other classes with more than half of all described extant species [1, 2]. Beetles (Coleoptera) encompass approximately 380,000 described species, representing ca. 40% of described insect diversity [3]. Several hypotheses have been proposed to explain this richness, notably their complex interactions with flowering plants [1, 4–7] and a high lineage survival rate [8]. Nevertheless, detailed supporting evidence from molecular genetic studies remains sparse, making it difficult to assess the relative importance of these and other potentially important contributing factors [9, 10].

The remarkable evolutionary success of beetles may have been driven by the interplay between their trophic niche and their genomic content and architecture. This is based on the premise that environmental and ecological conditions are likely to be predominant factors influencing the fate of genetic variation in populations under natural selection [11], eventually driving divergence into distinct species [12]. Among all components of the biotic environment, the trophic niche (principal source of nourishment) of an organism plays a crucial role in shaping the evolution of phenotypic innovations and their underlying genomic changes, e.g., feeding modes in cichlid fishes [13], mouth development in *Pristionchus* nematodes [14], and bitter taste receptors in vertebrates [15]. Among several hypotheses explaining the tremendous diversity among beetles, a shift from an ancestral diet as saprophages (detritus feeding) or mycophages (fungi feeding) [16] to phytophagy (feeding on living plant material in a broad sense) is often evoked [1, 4, 5]. While the suborder Adephaga (~ 45,000 species) comprises mostly predatory species, including ground beetles and diving beetles, the largest beetle suborder, Polyphaga (~ 315,000), is predominantly comprised of phytophagous clades, among which the most species-rich families are weevils (Curculionidae, ~ 51,000), longhorn beetles (Cerambycidae, ~ 30,000), and leaf beetles (Chrysomelidae, ~ 32,000) [3]. Phytophagy appeared approximately 425 million years ago, quickly after terrestrial life was established [17]. It progressively diversified to target most plant tissues [18], shortly before the radiation of flowering plants 120–100 million years ago [19]. In response, plants have evolved diverse strategies to protect themselves, which in turn impose selective pressures on the animals that feed on them.

While many biological processes are likely to play a role in this evolutionary battle, a key weapon in the arsenal of phytophagous insects’ adaptations is their ability to neutralize or minimize the effects of plant secondary compounds. Protein families known to be crucial for eliminating harmful plant toxins are cytochrome P450 monooxygenases (P450s), carboxylesterases (CEs), UDP-glycosyltransferases (UGTs), and glutathione S-transferases (GSTs) [20]. While P450s and CEs modify residues to make compounds more hydrophilic, UGTs and GSTs conjugate xenobiotic compounds to hydrophilic molecules. Detoxification is completed by membrane transporters, such as ATP-binding cassette (ABCs) transporters, which move xenobiotic compounds to where they can either be excreted or less frequently sequestered in order to be reused as a defense mechanism [20]. Additionally, to prevent phytophagous insects from digesting their tissues, plants produce enzyme inhibitors that block catalytic sites or compete with the substrates of enzymes involved in digestion. The major families affected are endopeptidases, such as cysteine (CYSs), and serine (SERs) proteases, as well as more specific enzymes such as glycoside hydrolases (GHs), certain types of which are able to break down polysaccharide molecules, including cellulose, hemicellulose, and pectin in plant cell walls [21, 22]. Other adaptations to phytophagy include repertoires of chemoreceptors that are crucial for finding appropriate food sources [23] and the specialization of mouthparts in response to plant mechanical barriers, which are highly diversified in insects [24].

As lineages diverge, their genomes accumulate changes, some of which are expected to be directly linked to functional adaptations. Identifying such genomic features and linking them to phenotypic differences, while robustly distinguishing between the effects of stochastic changes and natural selection [25], is critical to deciphering the genomic drivers of species radiations [26]. Changes include point substitutions, which may affect the existing functional elements, but also larger-scale changes such as duplications, from individual genes to entire genomes, which by adding new members to the repertoires of key gene families may constitute an ideal mechanism to facilitate the emergence of novel functions leading to successful phytophagy [27]. Whereas newly generated gene copies are usually redundant or deleterious and pseudogenized, rendering the gene copy non-functional [28], they are sometimes maintained. Particularly, interesting cases of gene family expansions are the ones restricted to specific lineages, resulting in lineage-specific expansion (LSE). Evolutionary mechanisms causing LSE are numerous and not all adaptive (see [28] for a comprehensive review). However, duplicated gene copies may provide an immediate selective advantage and be maintained by selection. This can be due to an increased dosage of the gene product, or to changes following the duplication being selected in one gene copy but not the other, which might allow evolution towards a different function in so-called neo-functionalization processes. Enzymes are considered particularly relevant candidates for such evolutionary processes as they could expand their range of substrates [29].

Here, we apply a comparative genomics approach to examine the evolution of genes putatively involved in plant-insect interactions by sampling from the two largest beetle suborders, which, generally speaking, present contrasting trophic niches. We contrast exemplars from the characteristically predaceous Adephaga with exemplars from Polyphaga, and we hypothesize that plant-insect interactions during the dietary shift to phytophagy should be accompanied by genomic evolutionary signatures visible at the subordinal scale. Using genomic and transcriptomic data from 18 beetle species, we estimate ancestral gene family content, taking into account gene gains and losses across the species phylogeny, to identify significant LSEs of gene families related to phytophagy and signatures of adaptive expansions in these families. Ignoring sensory receptors, as their evolution might be driven by agents other than those related strictly to trophic niche [30], and morphological genes, as their inferred association with diet is less robust, we focus on genes coding for enzymes, for which adaptive LSE specific to Polyphaga would suggest a role for detoxification and digestive pathways in driving adaptation and speciation.

## Results

### A representative sampling of the two major coleopteran suborders

**Fig. 1:**
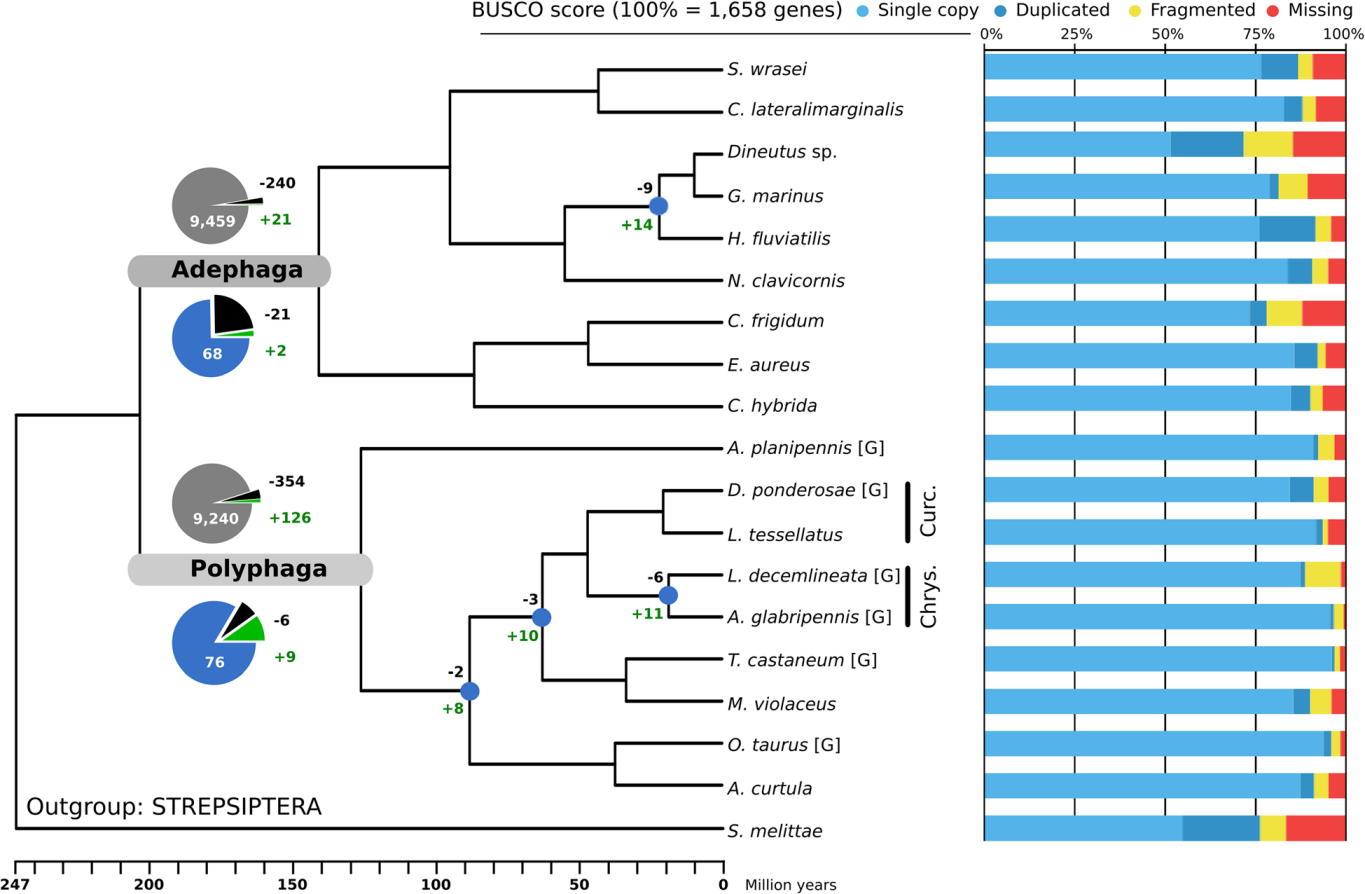
The ultrametric species phylogeny with gene family expansions and contractions quantified for nodes of interest and bar charts showing completeness of the genomic and transcriptomic datasets studied. The species tree was built from 405 single-copy orthologs and constrained to have Geadephaga (*C. frigidum*, *E. aureus*, *C. hybridia*) and Hydradephaga (the six other Adephaga) as monophyletic sister clades (e.g., following [6]). Branch lengths are scaled in millions of years. Maximum likelihood bootstrap support was 99 or 100% for all branches. [G] symbol indicates data from species with sequenced genomes with the remaining species being from transcriptomes. The numbers of orthologous groups (OGs) with expansions (+) and contractions (−) are displayed at the root node of each suborder. Pie charts show proportions of OGs with gene losses (black) and gene gains (green) with respect to OGs with no significant losses or gains for all considered OGs (gray) and only the candidate OGs (blue). While gains constitute only a small subset of all OGs in both suborders, the proportion of gains is much larger among candidate OGs in Polyphaga. The nodes indicated by blue circles in the Polyphaga subtree lead to species-rich clades containing species that are largely phytophagous (e.g., Chrysomelidae and Curculionidae, respectively Chrys. and Curc.) and experienced larger proportions of gains among the candidate OGs. The Benchmarking Universal Single-Copy Ortholog (BUSCO) scores indicate the relative levels of completeness and putative gene duplications for the genome-based and transcriptome-based datasets in terms of 1658 BUSCOs from the insecta_odb9 assessment dataset

Reliable estimation of gene gain and loss events requires a robust evolutionary framework, i.e., a phylogeny that includes the species studied, as well as the characterization of gene families across complete gene sets from these same species. To study adaptation to phytophagy, we sampled from both Adephaga (mostly predaceous) and Polyphaga (with diverse trophic habits, including a very large number of phytophagous species). A balanced sampling of each suborder was achieved comprising 12 transcriptomes and 6 genomes, with Benchmarking Universal Single-Copy Ortholog (BUSCO) completeness estimates [31, 32] ranging from 71.9 to 97% (Fig. 1, Table 1). The molecular species phylogeny estimated using protein sequences of 405 BUSCO genes found to be complete in all species and in the strepsipteran outgroup, *Stylops melittae*, was used to build the time-calibrated species phylogeny (Fig. 1). Subsequent analyses of gene gain and loss rates and of signatures of adaptive gene family expansion employed this ultrametric species tree, which importantly shows no significant difference in node ages within each suborder. Protein-coding sequence predictions ranged from 9844 to 24,671 genes per beetle species. These sequences matched 14,908 Arthropoda orthologous groups (OGs) containing at least 1 species of Coleoptera in the OrthoDB v8 catalog [40]. This represented a minimum of 6742 and a maximum of 11,149 OGs for *Carabus frigidus* and *Leptinotarsa decemlineata*, respectively. OGs containing genes from only 1 of the 2 sampled suborders were excluded, resulting in a total of 9720 OGs for the analysis that have evolutionary histories traceable to the last common ancestor of beetles. Functional annotations of the sequences within these OGs were used to identify and assign several of them to enzyme families relevant to the tested hypothesis. These candidate OGs comprised 4 UGTs, 22 P450s, 19 CEs, 6 GSTs, 4 SERs, 7 CYSs, 28 ABCs, and 1 GH, for a total of 91 candidate OGs from 8 families of genes (i.e., functional categories) (Table 2).

**Table 1:** Beetle genomes and transcriptomes included in the study. Taxonomic classifications are listed with data sources, as well as completeness (Benchmarking Universal Single-Copy Ortholog, BUSCO, score, C = complete, S = complete single-copy, D = complete duplicated, F = fragmented, M = missing), number of predicted proteins, and number of orthologous groups (OGs) with genes from each species. The outgroup species used in the phylogeny, *Stylops melittae*, belongs to the order Strepsiptera, which is the sister group of Coleoptera [33]

Species	Short form	Suborder	Family	Type of assembly	Accession	Bioproject	Source	BUSCO score (1658 genes)	Predicted proteins	OrthoDB groups (OG)
*Cicindela hybrida*	CHYBR	Adephaga	Carabidae	Transcriptome	GDMH01000000	PRJNA286505	1KITE, this study	C: 90.4 [S: 84.9%, D: 5.5%], F: 3.4%, M: 6.2%	13,916	8111
*Calosoma frigidum*	CFRIG	Adephaga	Carabidae	Transcriptome	GDLF01000000	PRJNA286499	1KITE, this study	C: 78.3% [S: 73.5%, D: 4.8%], F: 9.8%, M: 11.9%	9844	6742
*Elaphrus aureus*	EAURE	Adephaga	Carabidae	Transcriptome	GDPI01000000	PRJNA286520	1KITE, this study	C: 92.4% [S: 85.9%, D: 6.5%], F:2.2%, M: 5.4%	12,808	8020
*Noterus clavicornis*	NCLAV	Adephaga	Noteridae	Transcriptome	GDNA01000000	PRJNA286561	1KITE, Vasilikopoulos et al. [34]	C: 90.9% [S: 84.1%, D: 6.8%], F: 4.4%, M: 4.7%	12,981	7918
*Haliplus fluviatilis*	HFLUV	Adephaga	Haliplidae	Transcriptome	GDMW01000000	PRJNA286525	1KITE, Vasilikopoulos et al. [34]	C: 91.8% [S: 76.3%, D: 15.5%], F: 4.3%, M: 3.9%	19,408	8528
*Cybister lateralimarginalis*	CLATE	Adephaga	Dytiscidae	Transcriptome	GDLH01000000	PRJNA286512	1KITE, Vasilikopoulos et al. [34]	C: 88.2% [S: 83.1%, D: 5.1%], F: 3.7%, M:8.1%	13,916	7256
*Sinaspidytes wrasei*	SWRAS	Adephaga	Aspidytidae	Transcriptome	GDNH01000000	PRJNA286492	1KITE, Vasilikopoulos et al. [34]	C: 87.0% [S: 76.7%, D: 10.3%], F: 4.0%, M: 9.0%	13,392	7721
*Dineutus* sp.	DINEU	Adephaga	Gyrinidae	Transcriptome	GDNB01000000	PRJNA286516	1KITE, Vasilikopoulos et al. [34]	C: 71.9% [S: 51.6%, D: 20.3%], F: 13.6%, M: 14.5%	14,644	7089
*Gyrinus marinus*	GMARI	Adephaga	Gyrinidae	Transcriptome	GAUY01000000	PRJNA219564	1KITE, Misof et al. [35]	C: 81.5% [S: 79.0%, D: 2.5%], F: 8.1%, M: 10.4%	13,867	7663
*Aleochara curtula*	ACURT	Polyphaga	Staphylinidae	Transcriptome	GATW01000000	PRJNA219522	1KITE, Misof et al. [35]	C: 91.4% [S: 87.5%, D: 3.9%], F: 4.0%, M: 4.6%	20,280	8513
*Anoplophora glabripennis*	AGLAB	Polyphaga	Cerambycidae	Genome	GCF_000390285	PRJNA167479	I5k, McKenna et al. [21]	C: 96.9% [S: 95.8%, D: 1.1%], F: 2.7%, M: 0.4%	22,035	10,959
*Agrilus planipennis*	APLAN	Polyphaga	Buprestidae	Genome	GCF_000699045	PRJNA230921	I5k, unpublished	C: 92.5% [S: 91.2%, D: 1.3%], F: 4.5%, M: 3.0%	15,497	9089
*Dendroctonus ponderosae*	DPOND	Polyphaga	Curculionidae	Genome	GCF_000355655	PRJNA162621	Keeling et al. [36]	C: 91.2% [S: 86.0%, D: 5.2%], F: 4.1%, M: 4.7%	13,457	8518
*Leptinotarsa decemlineata*	LDECE	Polyphaga	Chrysomelidae	Genome	GCF_000500325	PRJNA171749	I5k, Schoville et al. [37]	C: 88.9% [S: 87.5%, D: 1.4%], F: 9.9%, M: 1.2%	24,671	11,149
*Laparocerus tessellatus*	LTESS	Polyphaga	Curculionidae	Transcriptome	10.5281/zenodo.1336288	N/A	This study, Seppey et al. [38]	C: 93.8% [S: 91.9%, D: 1.9%], F: 1.4%, M: 4.8%	18,448	8616
*Meloe violaceus*	MVIOL	Polyphaga	Meloidae	Transcriptome	GATA01000000	PRNJA219578	1KITE, Misof et al. [35]	C: 90.3% [S: 85.6%, D: 4.7%], F: 5.9%, M: 3.8%	14,295	8480
*Onthophagus taurus*	OTAUR	Polyphaga	Scarabaeidae	Genome	GCF_000648695	PRJNA167478	I5k, unpublished	C: 96.2% [S: 93.9%, D: 2.3%], F: 2.5%, M: 1.3%	17,483	9315
*Tribolium castaneum*	TCAST	Polyphaga	Tenebrionidae	Genome	GCF_000002335	PRJNA12540	Richards et al. [39]	C: 97.0% [S: 96.5%, D: 0.5%], F: 1.6%, M:1.4%	16,645	9429
*Stylops melittae*	SMELI	Outgroup	Stylopidae	Transcriptome	GAZM02000000	PRNJA219603	1KITE, Misof et al. [35]	C: 76.5% [S: 55.0%, D: 21.5%], F: 7.1%, M: 16.4%	13,026	6104

**Table 2:** Candidate gene categories with the keywords and identifiers used to select them from the full sets of sequences annotated with InterProScan. To be included as candidate orthologous groups (OGs) in the category, OGs were required to have at least one sequence matching both a UniRef and an InterProScan entry, and an additional gene ontology term in the case of serine proteases

Gene family category	InterProScan (Pfam or InterPro identifiers) or Gene Ontology	UnifRef keyword	Number of OGs
UDP-glycosyltransferases (UGTs)	PF00201	name:“cluster UDP glucuronosyltransferase” OR name:“cluster UDP glycosyltransferase”	4
Cytochrome P450 oxidases (P450s)	PF00067	name:“cluster Cytochrome P450”	22
Carboxylesterases (CEs)	PF02230, PF00135	name:“cluster carboxylesterase” OR name:“carboxylic ester hydrolase”	19
Glutathione S-transferases (GSTs)	PF00043, PF02798	name:“cluster Glutathione S-transferase”	6
Serine proteases (SERs)	PF00450, PF12146, PF05577, GO:0008236	name:“cluster Serine protease” OR name:“cluster Serine peptidase”	4
Cysteine proteases (CYSs)	PF00112	name:“cluster cysteine protease” OR name:“cluster cystein protease” OR name:“cluster Papain”	7
ABC transporters (ABCs)	PPF00005, PF00664	name:“cluster ABC”	28
Glycoside hydrolases (GHs)	IPR000334, IPR000743, IPR001360, IPR001547	name:“cluster Glycoside hydrolase”	1
Total			91

### Polyphaga exhibit more frequent gains across a larger set of OGs

Analysis of per-species gene counts of the complete set of 9720 OGs was performed with the Computational Analysis of gene Family Evolution (CAFE v3) [41] tool. CAFE analyses changes in gene family sizes using a stochastic birth and death process to model gene gain and loss across a species phylogeny. It estimates gain and loss rates from the extant gene count data, taking errors into account to allow for accurate inferences even with incomplete datasets, and identifies gene families with significantly accelerated rates of gain and loss. The mode considering distinct gene gain (*λ* = 0.0019 gain/gene/million years) and gene loss (*μ* = 0.0018 loss/gene/million years) was preferred over a single value for *λ* and *μ*, having a significantly greater maximum likelihood score (see the “Methods” section). The *λ* (gain) and *μ* (loss) values predicted when CAFE was run on each suborder separately were *λ* = 0.0020 and *μ* = 0.0027 for Adephaga, versus *λ* = 0.0023 and *μ* = 0.0021 for Polyphaga, showing a tendency for Adephaga to lose genes and for Polyphaga to gain genes. Among the 9720 OGs were 21 with reported expansions originating at the Adephaga root and 126 at the Polyphaga root (see Fig. 1 to locate the nodes). Conversely, 240 OGs showed gene losses for Adephaga and 354 for Polyphaga. Two expansions and 21 losses affected the candidate OGs for Adephaga, and 9 expansions and 6 losses for Polyphaga. Other polyphagan nodes leading to phytophagous-rich clades (i.e., Chrysomeloidea and Curculionidae) also exhibited more candidate OGs expanding than contracting (Fig. 1). All counts of gene gains and losses per node are presented in Additional file 1: Figures S1 and S2. Additionally, CAFE assigned individual OG *p* values of < 0.01 to a subset of 910 (9.3%) OGs, which, according to De Bie et al. 2006 [42], indicates gene families likely to have experienced accelerated rates of gain and loss. These are interesting to investigate further as they may represent large OGs of potentially unequal size between the suborders. Among these were 26 of the 91 candidate OGs (28.6%), a significantly larger proportion (two-sample test for equality of proportions, chi-square test, *p* value < 0.0001) compared with just 9.3% of non-candidate OGs.

### Signatures of adaptive expansion are more prevalent in Polyphaga

**Fig. 2:**
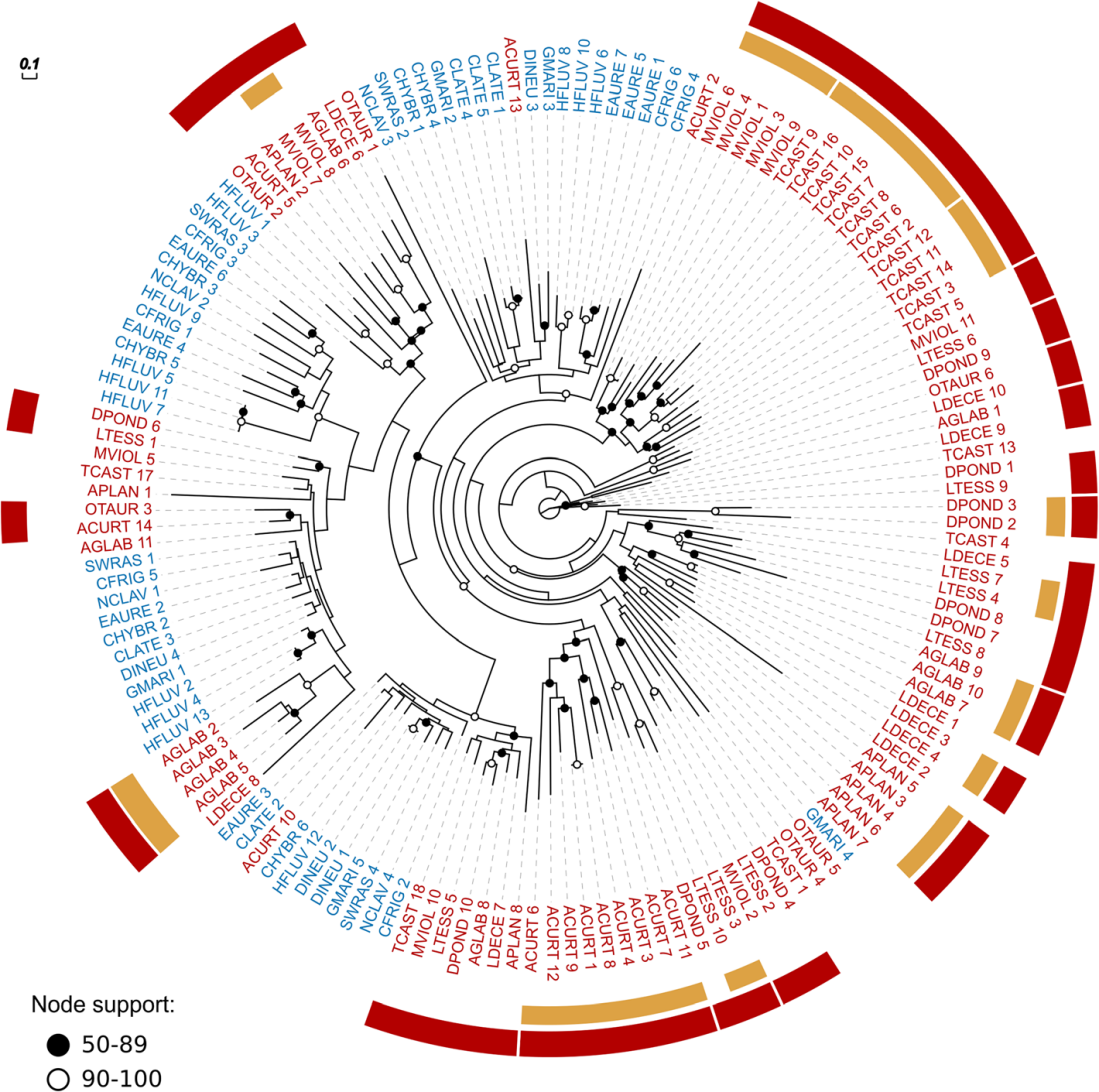
Molecular phylogeny from the largest glutathione S-transferase (GST) orthologous group among those exhibiting lineage-specific expansions driven by selection. Red labels indicate genes belonging to species of Polyphaga, accounting for 98 out of 152 genes (their Ornstein-Uhlenbeck per-species optimum is 11.69 vs. 6.85 for Adephaga (blue labels), see Table 3). The presence of several clades of polyphagan and adephagan genes delineates duplication events following the divergence of the two suborders. Encircling the gene labels are red bars that highlight polyphagan clades with bootstrap support of > 50% and yellow bars that highlight intra-specific duplications with bootstrap support of > 50%. Corresponding full names of species are given in Table 1. Branch lengths represent substitutions per site and bootstrap support below 50% is not displayed

All 910 OGs with significant variations in their gene content were tested for signatures of adaptive expansion in each suborder, by comparing Brownian motion (BM, neutral) to Ornstein-Uhlenbeck (OU, selective pressure) evolutionary models [43]. As mentioned previously, these included 26 OGs that belong to one of the functional categories listed in Table 2 (“candidate” OGs). The models consider per-species gene count as a trait that can evolve towards a value, which may or may not differ between the two suborders and may or may not be guided by selective pressure; we call this the “optimum” value in models integrating selection. The BM models assume no selection where differences between the suborders result from stochastic processes whose rates are estimated. The OU models assume that reaching an optimal gene family size in each suborder is driven by selective pressure. No adaptive LSE is represented by the null hypotheses of BM models with a single rate for the whole tree (BM1) or with different rates for each suborder (BMS) or the OU model with selection towards the same optimum for both suborders (OU1). Adaptive LSE is represented by the alternative hypotheses of OU models with selection towards two optima having the same variance (OUM) or with selection towards two optima having two variances (OUMV). In total, 21 OGs displayed a higher optimum for Adephaga (0.2% of the initial 9720 OGs) and 88 for Polyphaga (0.9%). Eight of these 88 OGs with higher optima in Polyphaga (Table 3; Additional file 1: Table S1; and gene trees in Fig. 2 and Additional file 1: Figures S3-S9) are candidate OGs belonging to one of the candidate gene families of Table 2, while none of the 21 OGs with higher optima in Adephaga belong to any of the candidate gene families. The proportion of OGs with expansions and higher optima in the background (all “candidate” and remaining “control” OGs) was significantly larger for Polyphaga compared to Adephaga (two-sample test for equality of proportions, chi-squared, 88/9720 vs. 21/9720, *p* value < 1e−09), indicating that Polyphaga have experienced globally more LSE under selection on protein-coding genes. Additionally, a test for enrichment (see the “Methods” section) of OGs with LSE under selection from the candidate families (Table 2) compared to the background was significant for Polyphaga (8/91 vs. 88/9720, *p* value < 1e−09). The same test applied individually on each candidate gene family within the candidate dataset demonstrated that categories enriched for LSE under selection in Polyphaga were GSTs (3/6 positive tests, FDR-corrected *p* value < 1e−09) and CEs (3/19, FDR-corrected *p* value < 0.005), as shown in detail in Table 4. Furthermore, functional genomics data from the polyphagan Asian longhorned beetle [21] supported a biological role in plant feeding activities for the candidate OGs that tested positive for adaptive expansions, which were enriched with genes upregulated in larvae fed on sugar maple trees vs. a nutrient-rich artificial diet (36/114 vs. 1391/12,461, *p* value < 1e−10).

**Table 3:** Candidate orthologous groups (OGs) with CAFE overall *p* values < 0.01 for which a model favoring selection for larger sizes in Polyphaga showed a greater likelihood. OG identifiers for functional category cytochrome P450s (P450), carboxylesterases (CE), glutathione S-transferases (GST), and cysteine proteases (CYS) are from OrthoDB v8 (ODB8 ID). Akaike Information Criterion corrected for small sample size (AICc) values are reported for all tested models. BM1 (Brownian motion with a single rate for the whole tree), BMS (Brownian motion with different rates for each regime), OU1 (selection towards the same optimum for both regimes) all represent the null hypothesis. OUM (selection towards two optima, same variance) and OUMV (selection towards two optima, two variances) represent the alternative hypotheses. The mean values in each suborder (Adephaga vs. Polyphaga) are presented in the last two columns. Values in italics indicate the preferred (maximum likelihood) model. A delta AICc > 2 is required for H1 to be retained

Category	ODB8 ID	BM1 AICc H0.1	BMS AICc H0.2	OU1 AICc H0.3	OUM AICc H1.1	OUMV AICc H1.2	Mean Adephaga	Mean Polyphaga
P450	EOG805VG7	148.37	153.21	143.35	148.10	*137.95*	34.13	34.47
CE	EOG87DCWX	143.23	143.77	143.63	*138.91*	141.15	6.55	18.78
CE	EOG8KD911	87.08	91.23	82.90	89.10	*79.74*	0.89	2.86
CE	EOG876NDC	80.64	85.67	80.08	87.42	*77.23*	1.72	3.48
GST	EOG87WR3Z	86.24	87.76	76.05	74.40	*72.12*	1.71	3.16
GST	EOG81RS7Z	108.77	114.44	109.19	113.76	*103.79*	6.85	11.69
GST	EOG85F05D	117.62	115.88	111.53	107.62	*106.32*	5.69	9.16
CYS	EOG8JDKNM	91.85	91.62	89.74	*85.66*	88.25	1.80	3.78

**Table 4:** Gene family category and candidate orthologous group (OG) enrichments among positive results. The top panel presents the statistical significance of each test for enrichment of candidate gene families among the positive results when compared to the background, for Polyphaga. The lower panel indicates the number of positive results in both suborders, for candidate OGs and background. Significant values at the 0.05 threshold are shown in italics

Category	Positive/total OGs	Category enrichment FDR
P450	1/22	0.36268
CE	3/19	*0.00252*
GST	3/6	*0.00016*
CYS	1/7	0.16627
UGT	0/4	1.00000
SER	0/4	1.00000
ABC	0/28	1.00000
GH	0/1	1.00000
Category	Positive/total OGs	Candidate vs. background enrichment *p* value
Background (Polyphaga)	88/9720	0
Candidates (Polyphaga)	8/91	
Background (Adephaga)	21/9720	1
Candidates (Adephaga)	0/91	

## Discussion

Comparative genomic analyses often highlight expanded gene families and link these expansions to biological functions peculiar to, or of special interest in, their focal organism(s). However, these analyses usually do not explicitly test for any hypothesized evolutionary model that might support such links. Here, we test a specific hypothesis of adaptation to a phytophagous diet, by comparing candidate gene family repertoires from nine adephagan (a mostly predaceous suborder) and nine polyphagan (a highly phytophagous suborder) beetle species. These candidate families are putatively involved in detoxification of plant allelochemicals and digestion of plant tissues. Specifically, we identify evolutionary signatures consistent with adaptive gene family expansions in the species-rich Polyphaga. These patterns should nevertheless be interpreted in the context of potentially confounding factors that could arise from combining genomic and transcriptomic datasets, conservative definitions of candidate gene families, or the greater species richness of the Polyphaga (see discussion points below and Additional file 1: Supplementary Results). Through explicitly testing for adaptive LSEs, these results offer support for the key evolutionary role of the phytophagous trophic niche in driving gene family expansions in Coleoptera (specifically Polyphaga), a feature that likely facilitated the adaptation of polyphagan beetles to specialized plant feeding.

### Dataset heterogeneity

For the comparison of gene repertoires between the two groups to be unbiased, the gene content of all analyzed species should be of similar accuracy and completeness. The number of predicted proteins for the genomic resources for each beetle species (Table 1, mean 15,977 and standard deviation 3748) was within the range expected for insects (see [44]). The average total gene count for Adephaga species (all transcriptomes) was about 4200 fewer than for Polyphaga, which include 2 genomes with more than 22,000 genes. This difference in average gene counts is reduced to just 1384 when considering only genes assigned to the 9720 OGs selected for the analysis. Our conservative orthology filtering therefore ensured that the comparisons focused on gene families with reliably traceable evolutionary histories that span both groups of beetles. Secondly, assessments of completeness showed that the majority of the datasets contained more than 90% of complete BUSCOs (Fig. 1, Table 1). While the dynamically evolving families that are the focus of this study are clearly not universal single-copy orthologs, the high levels of BUSCO completeness support the assumption that the datasets represent good coverage of the species’ gene content. Furthermore, the transcriptomes were sequenced from adults collected from their natural habitats, so members of the gene families that are the focus of our study were likely being actively transcribed at the time of sampling. Re-analyses of our data that exclude the two adephagan beetle species with fewer than 80% complete BUSCOs reduced the power of the model tests but nevertheless still identified the three GST OGs that favor a model with higher optima for Polyphaga (see Additional file 1: Table S2). Three of the adephagan transcriptomes showed more than 10% of duplicated BUSCOs, which could have arisen from suboptimal filtering of the transcriptomes, i.e., failure to remove alternative transcripts of the same gene. While such potentially inflated gene counts for these adephagans might prevent the identification of some true expansions in Polyphaga, they do not invalidate those that were identified. Conversely, transcriptome assemblies might collapse very similar paralogs into a single transcript and thereby underestimate true gene counts. Our gene trees (Fig. 2 and Additional file 1: Figures S3-S9) nevertheless show several examples of closely related paralogs from the transcriptomes, indicating that they can be successfully recovered. Finally, half of the OGs representing positive results showed a higher mean value for polyphagan transcriptomes than genomes, including the three GST OGs (Additional file 1: Table S1), and explicitly testing for effects due to using both genome and transcriptome data for the species of Polyphaga, by performing a modified OUwie analysis with data type as the regime under selection, identified only one CE (EOG8KD911) for which the favored model linked gene family expansion to species with genomes (see Additional file 1: Table S3).

### Candidate OG identification

The annotation strategy was designed to link OGs to candidate gene families based on manually selected keywords used to filter sequence search results, as well as Pfam and InterPro identifiers (Table 2), with the aim of excluding false positives (see the “Methods” section). This conservative strategy may not have fully captured all possible candidate OGs, which would therefore have remained in the background set of OGs that were used as controls. For example, we identified nine GST OGs (six were retained as candidates after filtering) while ten subclasses have been identified in arthropods [45]. While the strict (conservative) strategy we employed to identify candidate OGs may have resulted in an underestimate of the extent of the observed effects, this does not invalidate those that were identified. In addition, filtering the OGs to retain only those with genes from both Adephaga and Polyphaga excluded from the analyses any genes that were specific to either suborder. These might include genes with key roles in phytophagy, e.g., enzymes acquired by horizontal gene transfer identified from the *A. planipennis*, *A. glabripennis*, and *D. ponderosae* genomes [21]. While acknowledging their importance, here, we explicitly tested for adaptive LSE in one lineage vs. the other so gene evolutionary histories were required to span the two suborders and thus be traceable to their last common ancestor.

### The more speciose Polyphaga exhibit more dynamic gene repertoire evolution

The loss (*μ*) and gain (*λ*) values reported by CAFE on all 9720 OGs are consistent with assessments of other insect clades [46, 47]. Although the overall gain rate is slightly higher than the loss rate, the number of OGs losing genes reported by CAFE at each individual node is generally larger than the number of OGs with gains. This is reconciled by considering that across Coleoptera, many OGs lost a few genes while few families gained many genes. As most OGs display a low number of genes per species, i.e., they are evolving under “single-copy control” [48], losing more than one ortholog per species is understandably rare, while there is no theoretical limit for an OG to gain new members. Comparing the two clades, Polyphaga has a higher rate of gene gain and six times more OGs with gains, and while the Adephaga rate of gene loss is higher, Polyphaga have 1.5 times more OGs that have experienced gene losses. Importantly, these rates were estimated using a time-calibrated ultrametric species phylogeny with no significant difference in node ages between the two suborders. Hence, the gene repertoires of Polyphaga exhibit a more dynamic evolutionary history with more gains (rate) in more OGs (counts) and fewer losses (rate) spread out over more OGs (counts). It is possible that this greater dynamism may be generally linked to the greater species richness of Polyphaga, with no specific role for phytophagy underpinning this trend. However, among the candidate OGs for detoxification and digestion, there are also more gains in Polyphaga and, in contrast to the background, fewer losses. Thus, both gain and maintenance are higher for candidates in Polyphaga, which is consistent with a key role for phytophagy in driving dynamic gene repertoire evolution, and particularly LSEs.

### Support for adaptive expansions of gene families involved in detoxification in polyphagan beetles

In addition to observing more expansions among candidate OGs in the suborder Polyphaga, the positive results from the OUwie analysis support the hypothesis that selective pressures drive detoxification enzymes towards larger gene family sizes. This is especially pronounced for GSTs, for which half of the OGs tested positive and for which a significant enrichment compared to the background was found. The importance of GSTs in dietary shifts to phytophagy has been noted in mustard-feeding flies, where duplicated GSTs involved in the mercapturic acid pathway showed signatures of positive selection [49]. Our results therefore suggest that comparable phenomena have been acting at the level of polyphagan beetles. The CEs also show a statistically significant enrichment for positive results compared to the background, further supporting the diet detoxification hypothesis. The other positive results include a P450 OG and a CYS OG, neither of which led to a category enrichment compared to the background. The P450 OG is by far the largest among the positive results (Additional file 1: Figure S3), highlighting the importance of P450s in beetle (and generally insect) physiology with diverse roles beyond detoxification, e.g., hormone biosynthesis [50]. However, while considered as significantly expanded and under selection by our model, the actual mean values in the suborders are not dramatically different. Importantly, the enrichment of positive results among candidates still holds if this OG is excluded (see Additional file 1: Supplementary Results). The involvement of P450s in many other processes may explain why a broader difference between the suborders was not identified. Apart from one positive result among the cysteine proteases (no significant category enrichment), our study did not highlight additional expansions in other digestive enzymes or in transporters within a suborder. The lack of evidence for expansion in Polyphaga with respect to ABC transporters, which is the candidate functional category encompassing the largest number of OGs, may indicate that the ancestral diversity of transporters was sufficient for maintaining the excretion of toxins, despite variations in the substrates imposing a selective pressure on early stages of the detoxification pathway. Alternatively, if such pressure were acting on later stages of the pathway, i.e., transporters, its strength could have been too low for the detection power of our methods and data, unlike for GSTs or CEs.

## Conclusions

Our modeling of gene repertoire evolution across 18 beetle species identifies putative adaptive lineage-specific gene family expansions that accompany a dietary shift towards plant feeding. As discussed above, the use of transcriptome data for 12 species presents a potentially confounding factor that we attempt to control for in our analysis design and supplementary analyses. In addition, our set of species allows for only a single comparison between a mainly plant feeding and a mainly predacious suborder of beetles. Confirmation and generalization of our observed trends would thus ideally involve whole-genome sequencing to assemble and annotate high-quality genomes for improved resolution and confidence, as well as sampling from other beetle clades or some of the many groups of insects with dietary shifts towards plant feeding [51] to enable phylogenetic replication [52].

By comparing the degree of expansion among the gene families involved in detoxification of plant secondary compounds in two suborders of beetles characterized by generally contrasting trophic niches (i.e., Polyphaga contain a high proportion of phytophagous species while Adephaga encompass mostly predacious species), we identify genomic support for the popular hypothesis that Coleoptera species richness may be in part explained by their interaction with land plants. Candidate OGs of GSTs, CEs, P450s, and CYSs tested positive for adaptive LSEs in the phytophagous polyphagan beetle lineage, and categories of GSTs and CEs, in particular, were enriched for OGs with such adaptive LSEs. Moreover, across all OGs tested, Polyphaga exhibited significantly more adaptive LSEs than Adephaga. This indicates that genes other than the candidate detoxification and digestion enzymes, which could include genes with functions less obviously related or unrelated to phytophagy, are also likely to have played a role in the adaptive success and diversification of Polyphaga. While this suggests that additional functional categories remain to be explored, contrasting gene family evolution across the two major suborders of beetles suggests a role for interactions with plant secondary compounds, and supports a role for phytophagy in general, as important drivers of the remarkable radiation of polyphagan beetles.

## Methods

### Data sources

This study included 6 genomes and 13 transcriptomes representing a balanced sampling of polyphagan and adephagan beetles, along with 1 representative of the sister group to Coleoptera, Strepsiptera, to root the species phylogeny. Annotated gene sets from 4 genomes were sourced from the i5k pilot project datasets [53] (*Anoplophora glabripennis* v0.5.3 [21], *Leptinotarsa decemlineata* v0.5.3 [37], *Onthophagus taurus* v0.5.3, *Agrilus planipennis* v0.5.3) and 2 were independently published *Dendroctonus ponderosae* Ensembl Metazoa v1.0 [36] and *Tribolium castaneum* Ensembl Metazoa v3.22 [39]. One Polyphaga transcriptome, *Laparocerus tessellatus* (Additional file 1), was sequenced for this project [38], and the others were provided by the 1KITE project (Additional file 1, [34, 35, 54]). A detailed list is presented in Table 1.

### Coding sequence predictions, transcriptome, and genome quality assessments

Coding sequences and peptide sequences were predicted from all transcriptomes using TransDecoder (v2.0.1 https://transdecoder.github.io [last accessed May 8, 2019]) along with a custom python script to retain the best-scoring entry among overlapping predictions. The coding sequences and peptide sequences from the genomes were retrieved from their official annotated gene sets. All genome and transcriptome gene sets were assessed using BUSCO (v2.0, python 3.4.1, dataset insecta_odb9/2016-10-21, mode proteins) [32]. This tool identifies near-universal single-copy orthologs by using hidden Markov model profiles from amino acid alignments. CD-HIT-EST v4.6.1 [55] was run on the protein sequences with a 97.5% identity threshold to ensure that all species datasets were filtered to select a single isoform per gene.

### Orthology delineation

The OrthoDB [40] hierarchical orthology delineation procedure was employed to predict orthologous protein groups (OGs). Briefly, protein sequence alignments are assessed to identify all best reciprocal hits (BRHs) between genes from each pair of species, which are then clustered into OGs following a graph-based approach that starts with BRH triangulation. The annotated proteins from the genomes of *A. planipennis*, *O. taurus*, and all transcriptomes were mapped to OrthoDB v8 at the Arthropoda level (with 87 species including 4 of the beetles with sequenced genomes). Mapping uses the same BRH-based clustering procedure but only allows genes from mapped species to join existing OGs. These OGs were then filtered to identify the 9720 OGs with representatives from both Polyphaga and Adephaga to focus the study on OGs with evolutionary histories traceable to the last common ancestor of all the beetles, i.e., 5188 OGs with genes from only 1 of the 2 suborders were removed.

### Time-calibrated species phylogeny

To build an ultrametric phylogeny required for the CAFE analyses, the maximum likelihood molecular species phylogeny was first estimated based on the concatenated superalignment of orthologous amino acid sequences from each of the datasets. Protein sequences of single-copy BUSCO genes and the best-scoring duplicated genes present in all species were individually aligned for each set of BUSCO-identified orthologs using MAFFT with the --auto parameter [56], and each result was manually reviewed to exclude poor-quality alignments. Four hundred and five alignments were retained out of 436 and concatenated into a superalignment, partitioned according to the best model for each set of orthologs using aminosan 1.0.2015.01.23 [57]. RAxML v8.1.2 (-f a -m PROTGAMMA -N 1000) [58] was used to compute the maximum likelihood tree. The monophyly of Geadephaga and Hydradephaga was constrained to match the generally accepted resolution of Adephaga (as [6]). The chronos function of the R package ape (v3.4 on R 3.2.1, relaxed model) [59] was used to obtain an ultrametric tree, and the tip to root length was adjusted to match the approximately 250 million-year evolutionary history of crown group Coleoptera [6].

### Functional annotation and definition of candidate genes

InterProScan was run on all species' protein sets (-appl Pfam --goterms, 5.16.55) [60] to identify protein families. Additionally, blastp 2.3.0 [61, 62] was run against uniref50 (version June 22, 2016; [63]) with an *e* value cutoff of 1e−20. An OG was included in the set of candidate OGs when it had a match to both the uniref50 clusters and Pfam families [64] or gene ontologies [65] as detailed in Table 2.

### CAFE analysis

The number of genes in OGs for each species was counted. All candidates and remaining (control) OGs were pooled together and processed with CAFE 3.1 [41], to infer gene family evolution in terms of gene gains and losses. First, the python script provided by CAFE was used to estimate the error in our dataset. The CAFE software was then run using the mode in which the gain and loss rates are estimated together (*λ*) and a second mode in which they are estimated separately (gains = *λ*, losses = *μ*). The more complex model was retained as it reached a significantly better score (− 199,989 for a single estimated parameter and − 199,981 for two distinct estimated parameters, 2× delta log-likelihood = 16, chi-squared distribution, df = 1). For the entire analysis, the CAFE overall *p* value threshold was kept at its default value (0.01). To run CAFE on each suborder separately, the newick file was pruned to retain only the required species using newick utils 1.1.0 [66].

### Evolutionary models

To evaluate adaptive OG expansion, the likelihood of the count data was tested by optimizing parameters considering two methods provided by the OUwie R package v1.51 [43, 67]. First, a Brownian motion (BM) approach was used, which assumes no selection and thus differences result from a stochastic process whose rate is estimated. Second, Ornstein-Uhlenbeck (OU) models were used. They take into account an optimal family size that is obtained by selective pressure. Two groups were defined in the phylogeny, namely Polyphaga and Adephaga, to which the two different regimes to consider were assigned, plus a third regime to the root. This represents a simplified scenario allowing for the comparison of gene contents between one group and the other rather than attempting to estimate “levels” of phytophagy or zoophagy across the phylogeny. The models BM1 (Brownian motion with a single rate for the whole tree), BMS (Brownian motion with different rates for each group), and OU1 (selection towards the same optimum for both groups) were optimized as null hypotheses (H0) and compared to OUM (selection towards two optima, same variance) and OUMV (selection towards two optima, two variances) models as alternative hypotheses (H1). The Akaike information criterion corrected for small sample size (AICc) [68] was used to compare the models, and an AICc > 2 between the best H0 and the best H1 model was considered as significant to prefer the H1 model.

### Statistical enrichment

All results for candidates and controls were pooled together to obtain a background distribution of positive and negative results. Positive results are those OGs that passed the OUwie analysis, and negative results are all of the 9720 OGs that did not obtain a significant overall CAFE *p* value or did not pass the OUwie analysis. Then, 100,000 random draws (using the R function sample, without replacement) having the sample size of the candidate category to test for enrichment were taken from the background, and the significant outcomes for Polyphaga and Adephaga were counted. A *p* value was calculated for each group as follows: the number of random draws reaching the amount of significant outcomes found for the candidate category, or more, divided by 100,000. Additionally, the multiple tests conducted on each individual candidate category were corrected for false discovery rate (FDR) using the R p.adjust function (method BH, Benjamini Hochberg).

### Gene trees

The alignments for the gene trees for the eight OGs that tested positive for adaptive expansions were produced using MAFFT with the --auto parameter. The gene trees were computed with RAxML v8.1.2 (-f a -m PROTGAMMALGF -N 100) and plotted with EvolView [69, 70].

Further methods details are presented in Additional file 1: Supplementary Methods, and a chart summarizing the main steps of the analysis is available in Additional file 1: Figure S10.

## Additional file

Additional file 1: Supplementary results and methods. Details of the results from supplementary analyses and additional details on materials and methods including supplementary Tables S1–S4 and supplementary Figures S1–S10. (PDF 2228 kb)

## Abbreviations

ABC: Adenosine triphosphate-binding cassette transporters
AICc: Akaike Information Criterion corrected for small sample size
BM: Brownian motion
BUSCO: Benchmarking Universal Single-Copy Ortholog assessment tool
CAFE: Computational Analysis of gene Family Evolution tool
CE: Carboxylesterase
CYS: Cysteine protease
FDR: False discovery rate
GH: Glycoside hydrolase
GST: Glutathione S-transferase
LSE: Lineage-specific expansion
MAFFT: Multiple Alignment using Fast Fourier Transform tool
OG: Orthologous group
OU: Ornstein-Uhlenbeck
P450: Cytochrome P450 monooxygenase
SER: Serine protease
UGT: Uridine 5′-diphospho-glycosyltransferase
