# Supplementary material for: Genomic signatures accompanying the dietary shift to phytophagy in polyphagan beetles
Source: Genome Biol. 2019 May 17;20:98. doi: 10.1186/s13059-019-1704-5 (PMC6525341; doi:10.1186/s13059-019-1704-5)
Supplement: Supplementary file 1 — Supplementary results and methods. Details of the results from supplementary analyses and additional details on materials and methods including supplementary Tables S1–S4 and supplementary Figures S1–S10. (PDF 2228 kb) [file 13059_2019_1704_MOESM1_ESM.pdf]

Genomic signatures accompanying the dietary shift to phytophagy in polyphagan beetles

Mathieu Seppey, Panagiotis Ioannidis, Brent C. Emerson, Camille Pitteloud, Marc Robinson-Rechavi, Julien Roux, Hermes E. Escalona, Duane D. McKenna, Bernhard Misof, Seunggwan Shin, Xin Zhou, Robert M. Waterhouse*, Nadir Alvarez*

Additional file 1

Supplementary Results

Supplementary Methods

Supplementary Tables S1-S4

Supplementary Figures S1-S10

Supplementary Results

Methodological validations

We conducted several validations to exclude technical biases inherent to the design of the analysis. To evaluate the strength of our positive results knowing that the two species with lower BUSCO scores belong to the Adephaga suborder, with the risk of incomplete counts increasing the observed effect, we excluded the two lowest-scoring species in terms of completeness (*Carabus frigidus* and *Dineutus* sp.). Re-analysis with CAFE and OUwie with this unbalanced dataset of seven Adephaga vs. nine Polyphaga showed that three out of the eight previously positive results still stood with this lower power. Interestingly, the three orthologous groups (OGs) were the three GSTs (Supplementary Table S2). The

global analysis remained statistically significant if only these three GSTs were considered as positive results while the rest of the candidates were considered as negative (3/91, p-value < 0.05). To assess model sensitivity to the mixture of genome and transcriptome data, we ran a modified OUwie analysis using only Polyphaga species with the sequencing type, i.e. genome and transcriptome, as the regime under selection. This approach removed the actual biological effect of the suborders, testing only an effect of the sequencing type. Out of the eight initial positive results, only one CE (EOG8KD911) favoured a model in which the family is significantly larger for species represented by a genome (Supplementary Table S3). Finally, we compared the node ages of both suborders, to rule out a significant difference that could have provided to one suborder more time to experience changes. A Wilcoxon rank sum test applied on both subordinal node ages was not significant (p-value = 0.72).

Supplementary Methods

Laparocerus tessellatus transcriptome

Two males and two females were collected in March 2015 in the Anaga peninsula, on the Island of Tenerife in the Canary Islands. RNA was extracted using the Qiagen kit RNeasy (Qiagen, Hombrechtikon, Switzerland), with an extra DNase treatment. Strand specific libraries were prepared individually with the TruSeq Stranded mRNA kit (Illumina, San Diego, CA, USA) and sequenced by the Lausanne Genomic Technologies Facility (http://www.unil.ch/gtf [last accessed April 21, 2018]) on an Illumina HiSeq paired-end 100bp protocol. The four samples were merged and corrected using fastq-mcf v1.04.636 (Aronesty, 2011). The transcriptome was assembled using Trinity v2.0.6 (Haas et al., 2013). Transcripts with an average coverage below 20 were discarded. We removed contaminating transcripts of human, bacterial and plant origin (hg18, GCF_000184155.1, Rice Genome Pseudomolecules Release 5, GCF_000008025.1, GCF_000196615.1, and GCF_000008865.1) using BLAT alignments (Kent, 2002). Finally, we retained only the longest isoform for each transcript as clustered by Trinity. This final dataset is available from Zenodo (Seppey et al., 2018) at https://doi.org/10.5281/zenodo.1336288.

1KITE transcriptomes

We included nine published and three previously un-published transcriptomes from the 1KITE project. Transcriptomes were sequenced from whole-organism samples of wild-caught adults feeding in their natural habitats. The published data were downloaded from NCBI as Transcriptome Shotgun Assembly (TSA) (see taxon sampling and accession number details in Table 1 and additional details and contamination checks in Supplementary Table S4). The unpublished transcriptomes from 1KITE were sequenced following the methods of Misof et al., 2014, Peters et al., 2017, and Vasilikopoulos et al., 2019. Briefly, the samples were preserved in RNAlater and RNA was extracted using TRIzol (Invitrogen, Grand Island, NY, USA). The indexed paired-end cDNA library was constructed for transcriptome sequencing using Illumina HiSeq 2000 paired end 150bp (Illumina, San Diego, CA, USA). To assemble raw data, the SOAPdenovo-Trans-31kmer version 1.01 (Xie et al., 2014) was used. Further details about quality control and contamination checks for sequences are described in Peters et al., 2017.

Anoplophora glabripennis sugar maple feeding

Diet-dependent regulation of gene expression was previously investigated in the Asian longhorned beetle (AGLAB) employing an RNA-seq-based differential expression analysis of larvae feeding on the wood of living sugar maple trees (a preferred host) versus an artificial diet versus (McKenna et al., 2016). A total of 1391 genes from the background of 12461 genes from the filtered OGs used as input for the CAFE analysis were up-regulated in larvae feeding on sugar maple wood (11.16%). From the 8 OGs that tested positive for adaptive expansions in Polyphaga, 36 of the 114 AGALB genes were up-regulated in larvae fed on sugar maple wood (31.58%). A chi-squared 2-sample test for equality of proportions with continuity correction showed a significant enrichment of up-regulated genes from OGs that tested positive for adaptive expansions (p-value = 2.185e-11).

Node age

The node ages of both suborders were extracted using the R function ‘nodeHeights’ in the package phytools (Revell, 2012) and tested for a statistically significant difference using the R function wilcox.test.

Phylogeny pruning for methodological validations

To obtain trees containing only a subset of species, the newick file was pruned with newick utils 1.1.0 (Junier and Zdobnov, 2010) to select only the required leaves and branches.

Computing resources

BLAST, InterProScan, CAFE and BUSCO runs were performed on the SIB Swiss Institute of Bioinformatics Vital-IT cluster in Lausanne (http://www.vital-it.ch [last accessed August 6, 2017]) (R version 3.1.1, Python 2.7.5). The *L. tessellatus* transcriptome assembly was conducted on an Ubuntu Server 14.04.3 LTS (R version 3.0.2, Python 2.7.5). All other steps were performed on a MacBook Air, OSX 10.7.5 (R version 3.2.1, Python 2.7.1).

Custom scripts

Custom scripts available from Zenodo at https://doi.org/10.5281/zenodo.2593899 include:

[1] ade_vs_poly_OUwie.R, R script for running OUwie analyses

[2] chronos.R, R script for building ultrametric species phylogeny

[3] exclude_technical_biais.R, R script genome-transcriptome vs. Adephaga-Polyphaga

[4] stats.Rmd, code for statistical tests e.g. enrichments

[5] cafe directory, contains CAFE control files with all setting for running CAFE

Supplementary Tables

Supplementary Table S1. Per-species counts of genes for the eight positive results. Highest mean values are highlighted in bold.

**Species**	**Suborder**	**Type**	EOG 805VG7 P450	EOG 87DCWX CE	EOG 8JDKNM CYS	EOG 8KD911 CE	EOG 87WR3Z GST	EOG 81RS7Z GST	EOG 876NDC CE	EOG 85F05D GST
CHYBR	Adephaga	Transcriptome	32	8	1	0	1	6	1	1
CFRIG	Adephaga	Transcriptome	23	8	1	1	1	6	0	2
EAURE	Adephaga	Transcriptome	28	8	1	2	1	7	4	4
NCLAV	Adephaga	Transcriptome	33	4	1	2	1	4	0	3
HFLUV	Adephaga	Transcriptome	34	3	1	3	1	13	3	8
GMARI	Adephaga	Transcriptome	21	12	1	0	1	5	0	5
DINEU	Adephaga	Transcriptome	41	3	3	0	3	4	2	8
CLATE	Adephaga	Transcriptome	26	6	0	0	1	5	0	2
SWRAS	Adephaga	Transcriptome	15	7	1	3	1	4	1	3
APLAN	Polyphaga	Genome	6	8	1	5	1	8	2	2
TCAST	Polyphaga	Genome	46	16	3	3	3	18	4	7
LDECE	Polyphaga	Genome	37	30	9	5	6	10	2	3
AGLAB	Polyphaga	Genome	37	39	6	4	2	11	4	11
DPOND	Polyphaga	Genome	31	10	0	5	1	10	3	5
OTAUR	Polyphaga	Genome	30	33	1	4	2	6	1	12
LTESS	Polyphaga	Transcriptome	24	20	5	0	5	10	5	16
MVIOL	Polyphaga	Transcriptome	30	5	2	1	1	11	5	7
ACURT	Polyphaga	Transcriptome	16	8	1	2	3	14	1	4
Mean	Adephaga	Transcriptome	28.1	6.6	1.1	1.2	1.2	6.0	1.2	4.0
Mean	Polyphaga	Genome	**31.2**	**22.7**	**3.3**	**4.3**	2.5	10.5	2.7	6.7
Mean	Polyphaga	Transcriptome	23.3	11.0	2.7	1.0	**3.0**	**11.7**	**3.7**	**9.0**

Supplementary Table S2. Small-sample-size corrected Akaike Information Criterion (AICc) values for positive results when two adephagan species with low Benchmarking Universal Single-Copy Orthologue (BUSCO) completeness scores are excluded from the analysis. The glutathione S-transferases (GST) orthologous groups still favour a model with a higher optima for Polyphaga. NA denotes negative AICc. Bold values highlight the preferred model (delta AICc > 2 for H1 to be retained).

Category	ODB8 ID	BM1 AICc H0.1	BMS AICc H0.2	OU1 AICc H0.3	OUM AICc H1.1	OUMV AICc H1.2	Mean Adephaga	Mean Polyphaga
P450	EOG805VG7	**126.03**	131.60	128.60	137.07	125.09		
CE	EOG87DCWX	129.82	**128.43**	130.88	131.75	129.65		
CE	EOG8KD911	81.15	86.42	**75.88**	86.66	75.06		
CE	EOG876NDC	**70.89**	76.83	72.68	84.87	73.03		
GST	EOG87WR3Z	77.31	NA	70.43	NA	**67.06**	1.53	3.09
GST	EOG81RS7Z	99.34	105.40	100.04	107.92	**96.00**	7.40	11.8
GST	EOG85F05D	107.72	105.46	101.68	104.23	**98.36**	5.64	9
CYS	EOG8JDKNM	83.30	**67.91**	82.71	76.74	82.61		

Supplementary Table S3. Small-sample-size corrected Akaike Information Criterion (AICc) values for positive results when genome and transcriptome are considered as regime using only the data from the suborder Polyphaga. One out of the eight positive results is explained by genomes having larger values than transcriptomes. Bold values highlight the preferred model (delta AICc > 2 for H1 to be retained).

Category	ODB8 ID	BM1 AICc H0.1	BMS AICc H0.2	OU1 AICc H0.3	OUM AICc H1.1	OUMV AICc H1.2	Mean Genome	Mean Transcriptome
P450	EOG805VG7	**73.49**	77.95	85.45	91.01	79.88		
CE	EOG87DCWX	**78.32**	82.85	87.42	93.35	84.76		
CE	EOG8KD911	48.63	43.65	53.61	50.80	**38.83**	4.33	1.00
CE	EOG876NDC	**39.91**	44.23	50.31	61.36	50.15		
GST	EOG87WR3Z	**49.59**	54.24	53.09	64.91	52.91		
GST	EOG81RS7Z	**56.02**	60.46	64.74	72.74	64.09		
GST	EOG85F05D	**66.05**	69.29	70.21	81.24	69.63		
CYS	EOG8JDKNM	**52.88**	57.61	61.58	69.47	61.32		

Supplementary Table S4. Full details of all 1KITE transcriptomes, including contamination checks.

Species Name	1KITE Library ID	TSA accession	TSA version	BioSample Accession	Bioproject Accession	No. Contigs after assembly	After local VecScreen	After Contam. Check	No. Contigs published
Cybister lateralimarginalis	INSnfrTADRAAPEI-16	GDLH00000000	GDLH01000000	SAMN03799556	PRJNA286512	31,471	31,470	31,403	31,402
Dineutus sp.	INSbttTBRAAPEI-11	GDNB00000000	GDNB01000000	SAMN03799560	PRJNA286516	25,920	25,915	24,679	24,661
Gyrinus marinus	INSnfrTBERAAPEI-19	GAUY00000000	GAUY01000000	SAMN02047132	PRJNA219564	90,582	90,564	-	90,225
Haliplus fluviatilis	INShkeTBXRAAPEI-18	GDMW00000000	GDMW01000000	SAMN03799569	PRJNA286525	46,197	46,191	45,977	45,915
Noterus clavicornis	INShkeTALRAAPEI-37	GDNA00000000	GDNA01000000	SAMN03799605	PRJNA286561	21,719	21,716	21,606	21,601
Sinaspidytes wrasei	WHINSnuyTAAARAAPEI-47	GDNH00000000	GDNH01000000	SAMN03799537	PRJNA286492	41,855	41,748	37,769	37,371
Cicindela hybrida	INShauTBARAAPEI-21	GDMH00000000	GDMH01000000	SAMN03799549	PRJNA286505	26,377	26,377	26,286	26,286
Calosoma frigidum	INSbttTLRAAPEI-19	GDLF00000000	GDLF01000000	SAMN03799544	PRJNA286499	15,596	15,594	15,495	15,495
Elaphrus aureus	INShkeTBKRAAPEI-90	GDPI00000000	GDPI01000000	SAMN03799564	PRJNA286520	20,404	20,405	20,135	20,133
Aleochara curtula	INShauTBERAAPEI-33	GATW00000000	GATW01000000	SAMN02047128	PRJNA219522	52,043	52,033	-	51,642
Meloe violaceus	INShauTAYRAAPEI-19	GATA00000000	GATA01000000	SAMN02047163	PRJNA219578	20,135	50,610	-	50,507
Stylops melittae	INSytvTBKRAAPEI-43	GAZM00000000	GAZM02000000	SAMN02047139	PRJNA219603	20,508	20,507	19,975	19,820

Supplementary Figures

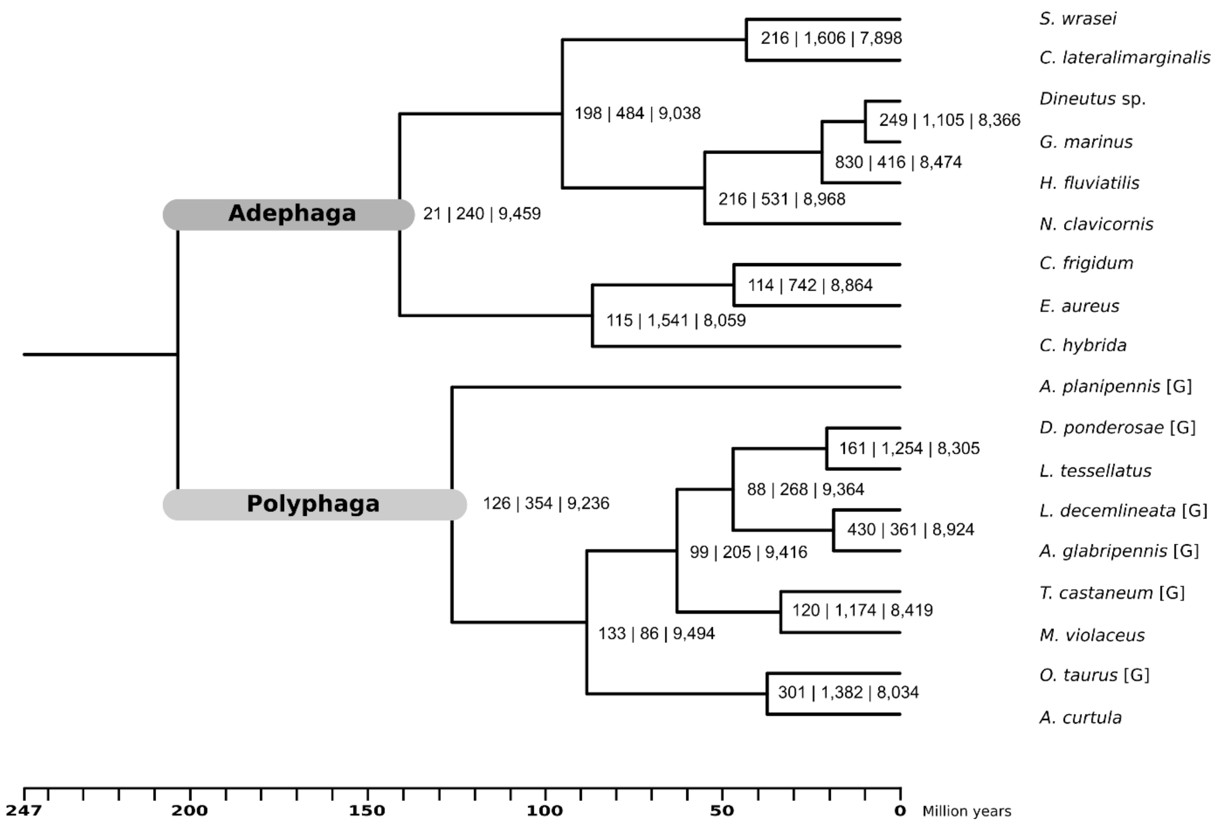

Supplementary Fig. S1. Detailed CAFE counts of expansion **|** contraction **|** unchanged, at each node, for the 9,720 orthologous groups (OGs) considered by Computational Analysis of gene Family Evolution (CAFE). [G] stands for species represented by a genome.

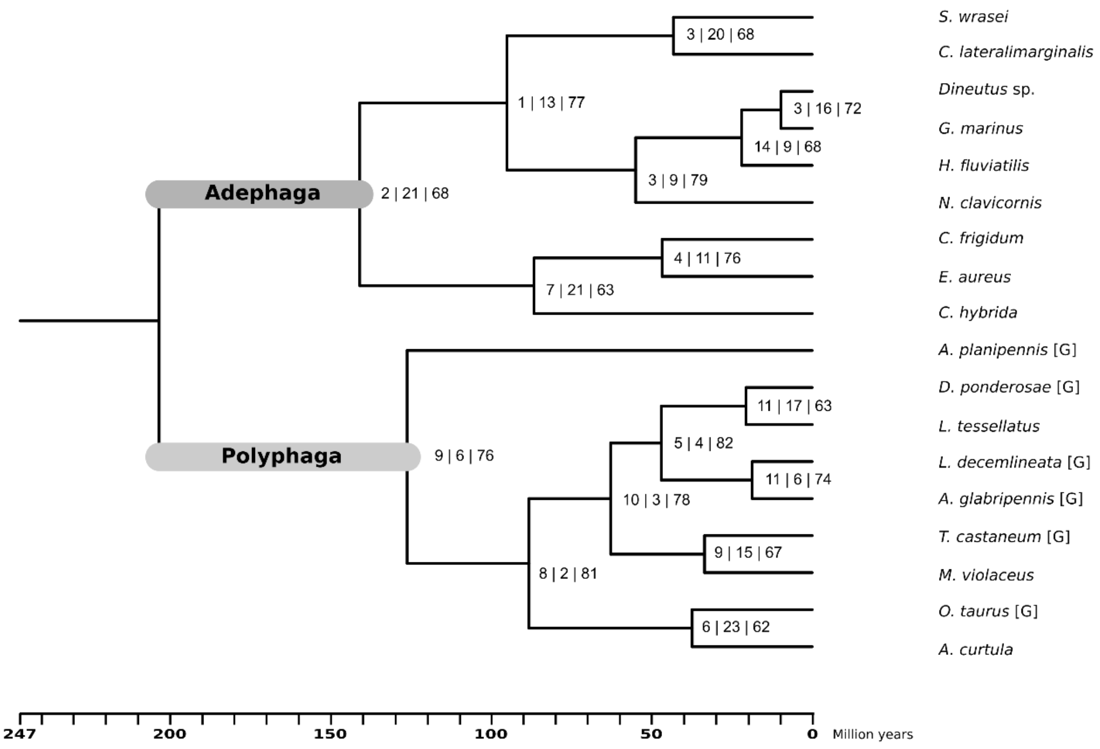

Supplementary Fig. S2. Detailed CAFE counts of expansion **|** contraction **|** unchanged, at each node, for the 91 candidate orthologous groups (OGs) considered by Computational Analysis of gene Family Evolution (CAFE). [G] stands for species represented by a genome.

Supplementary Fig. S3-S9. Molecular phylogenies representing each orthologous group (OG) included in positive results, in addition to Fig. 2. Red labels indicate genes belonging to species of Polyphaga and blue labels those belonging to species of Adephaga. Encircling the gene labels are red bars that highlight polyphagan clades with bootstrap support of >50% and yellow bars that highlight intra-specific duplications with bootstrap support of >50%. Corresponding full names of species are given in Table 1. Branch lengths represent substitutions per site and bootstrap support below 50% is not displayed.

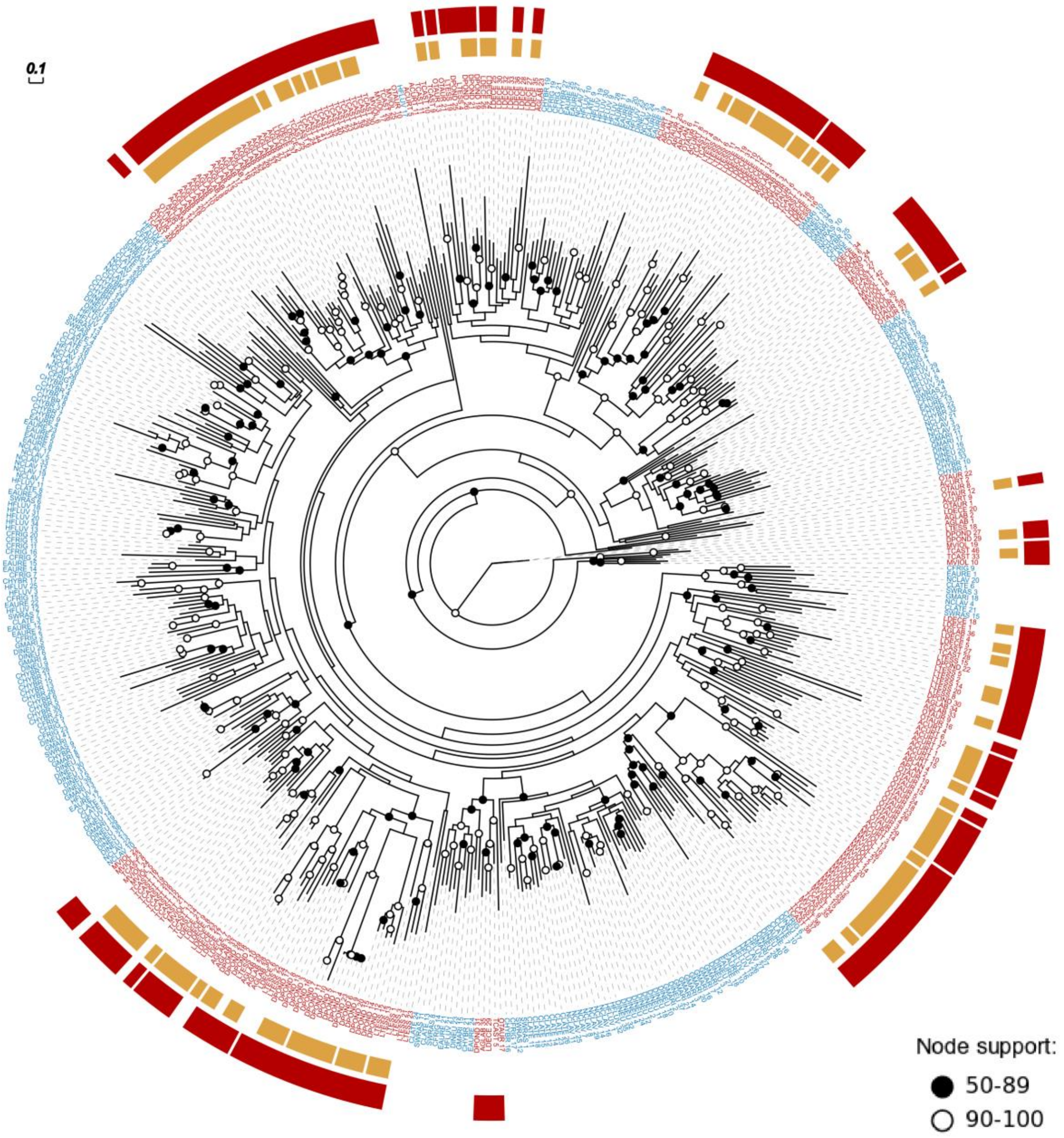

Supplementary Fig. S3. P450_EOG805VG

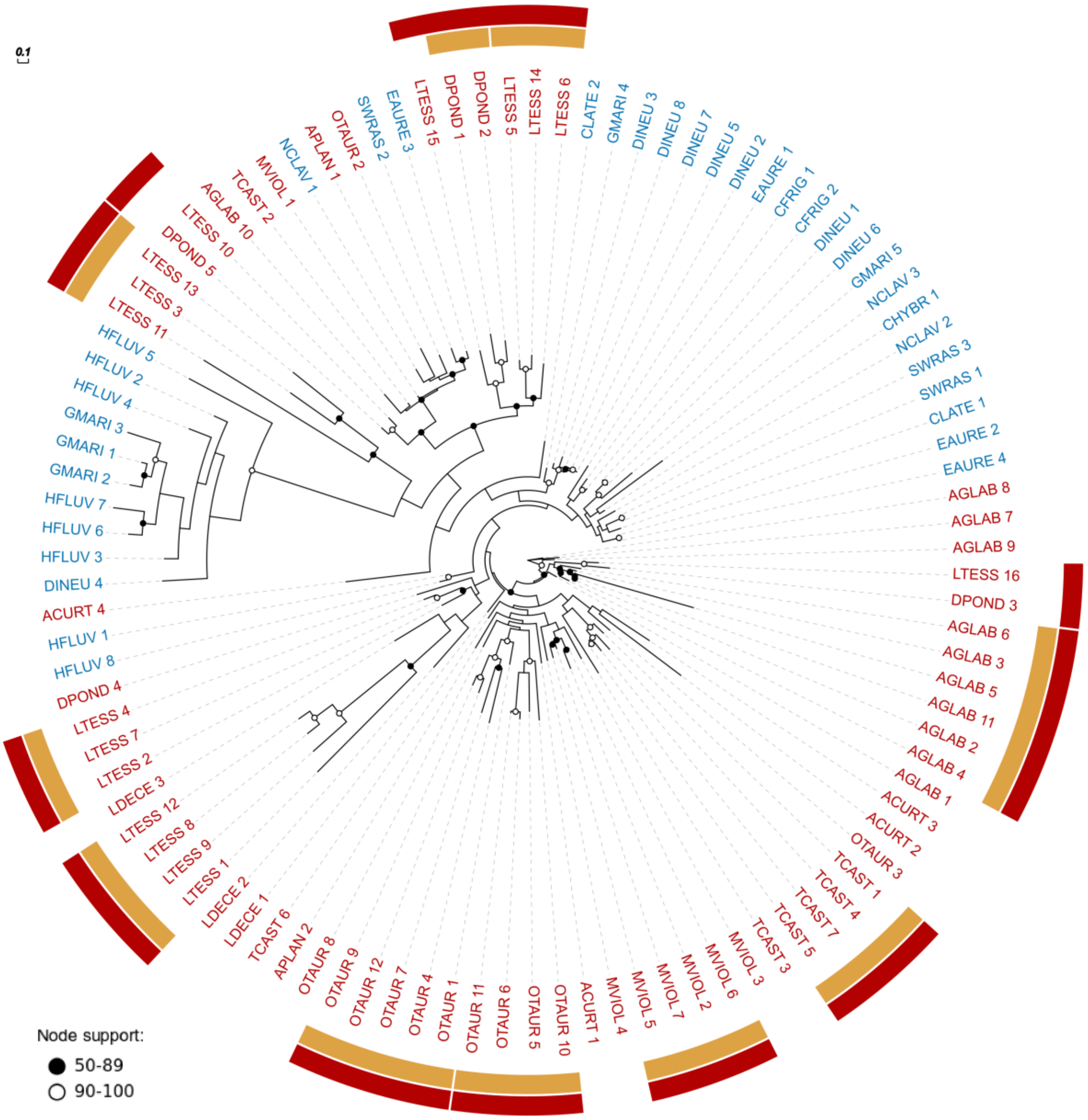

Supplementary Fig. S4. GST_EOG85F05D

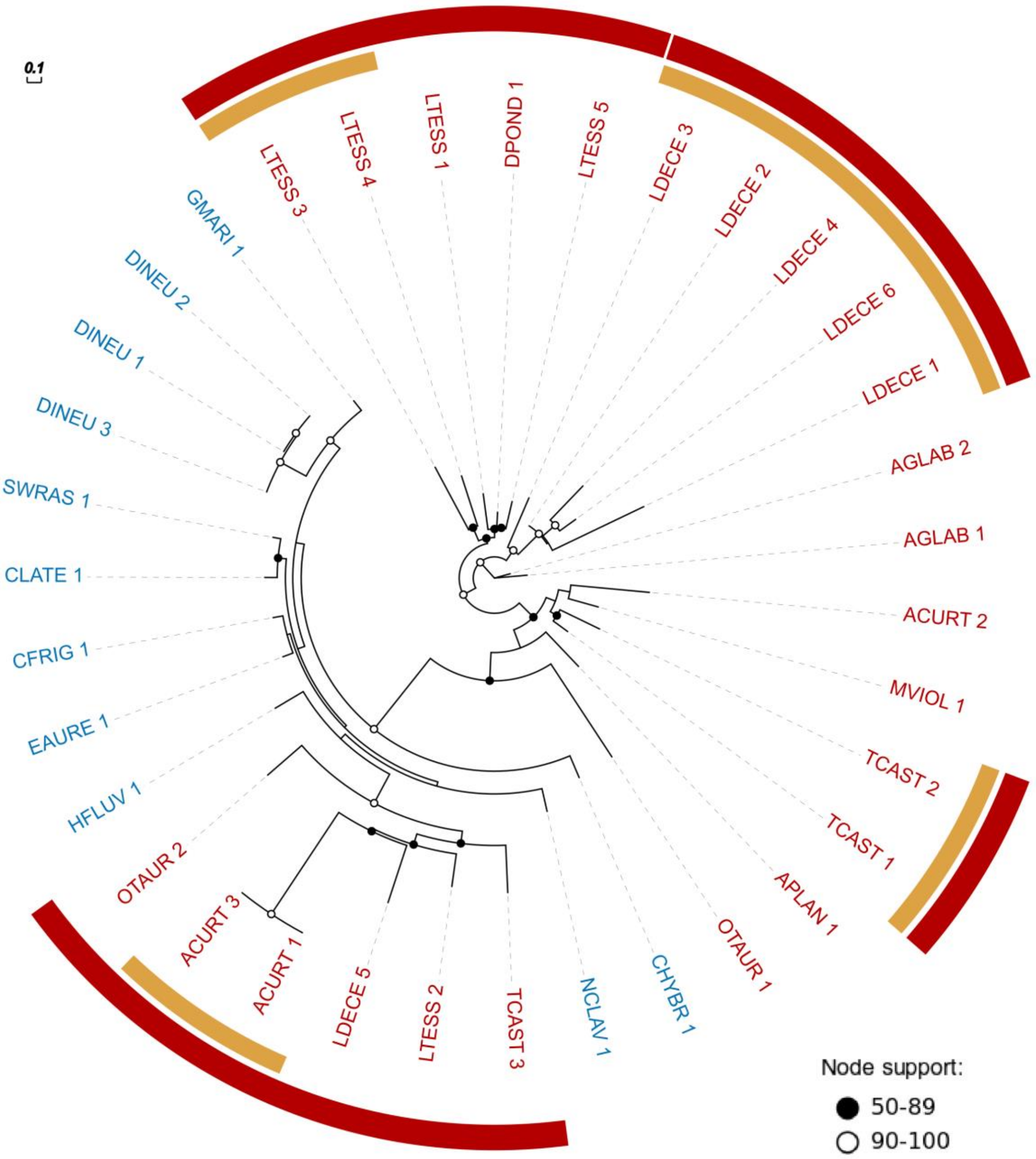

Supplementary Fig. S5. GST_EOG87WR3Z

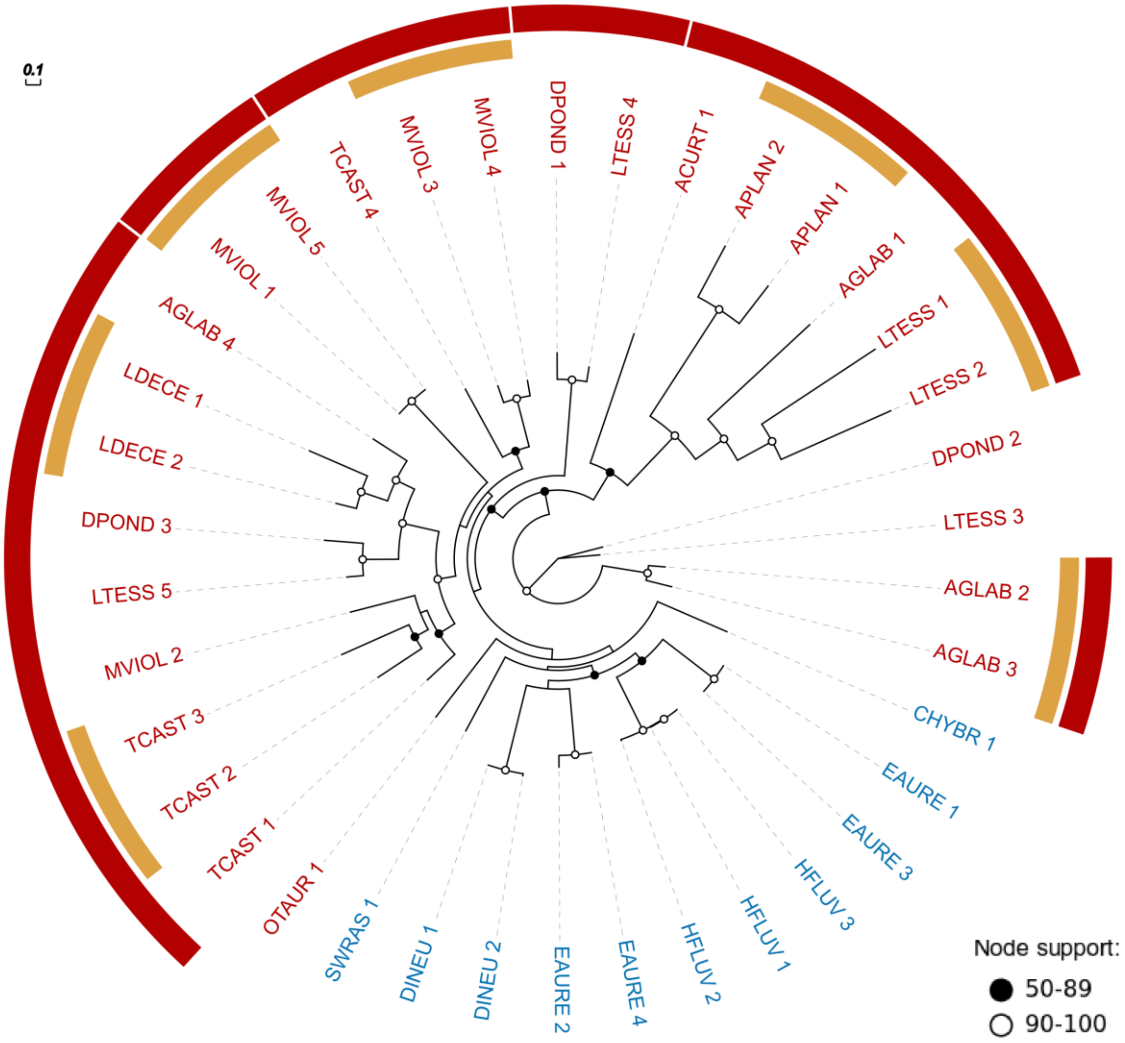

Supplementary Fig. S6. CE_EOG876NDC

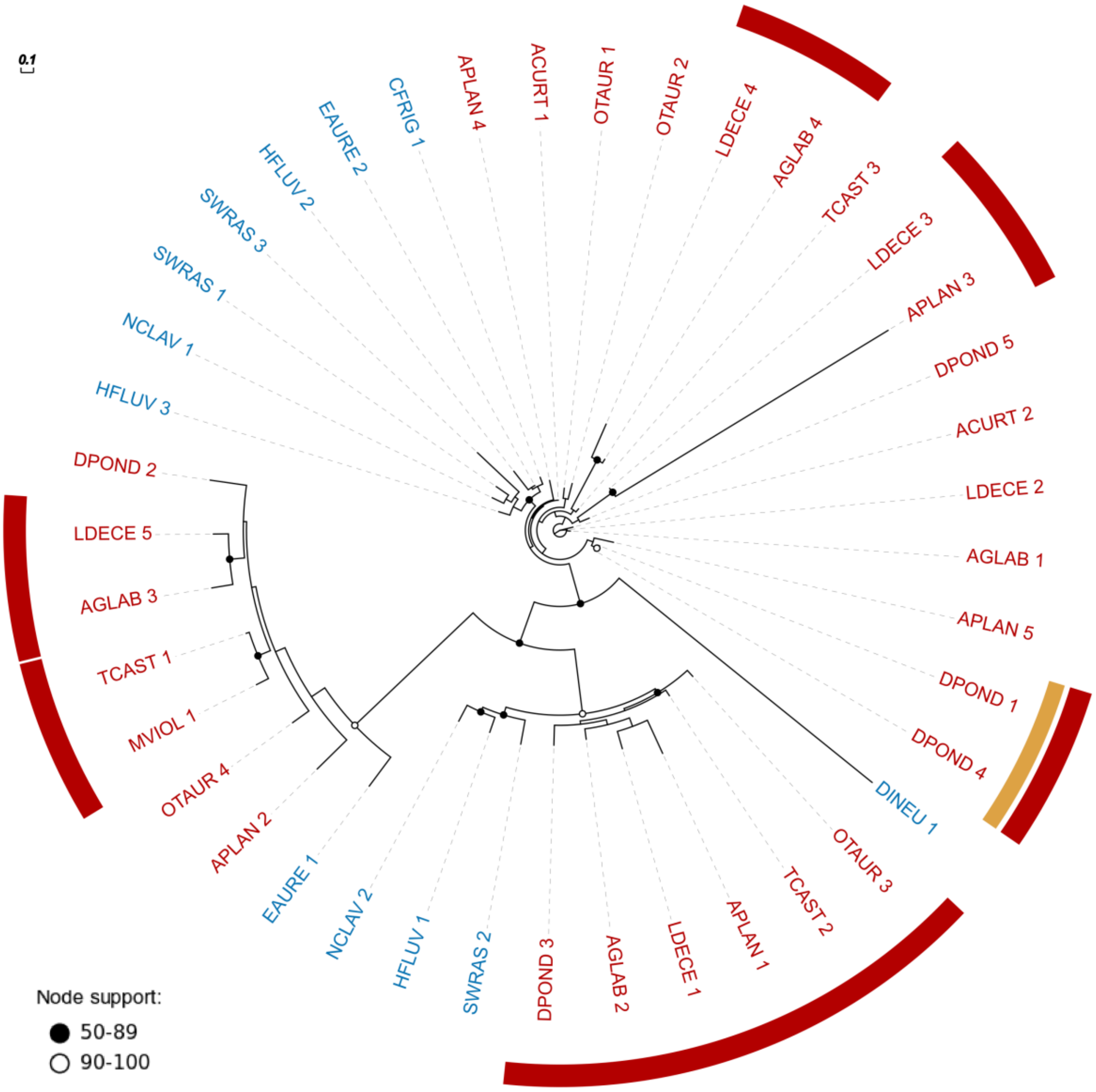

Supplementary Fig. S7. CE_EOG8KD911

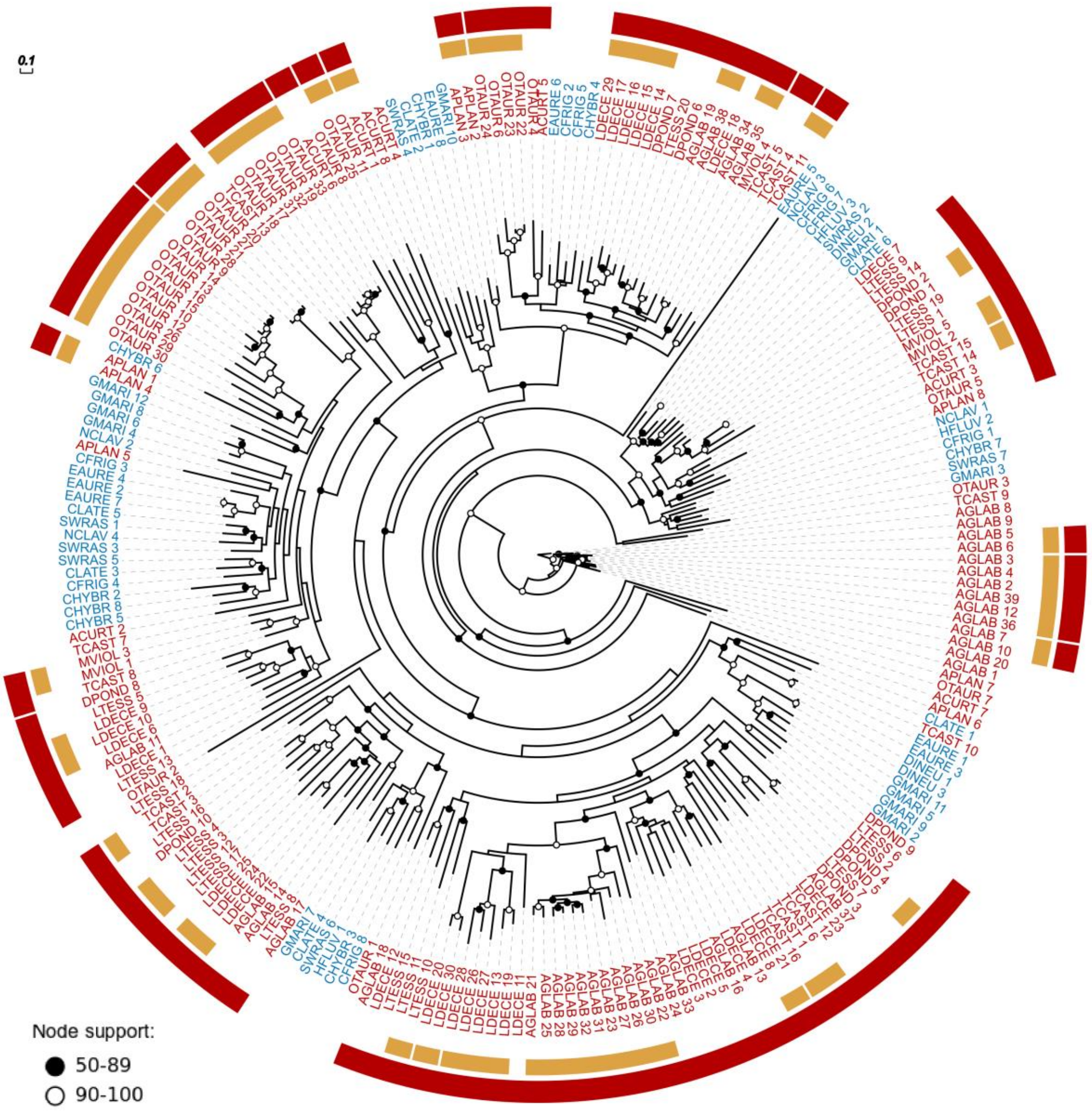

Supplementary Fig. S8. CE_EOG87DCWX

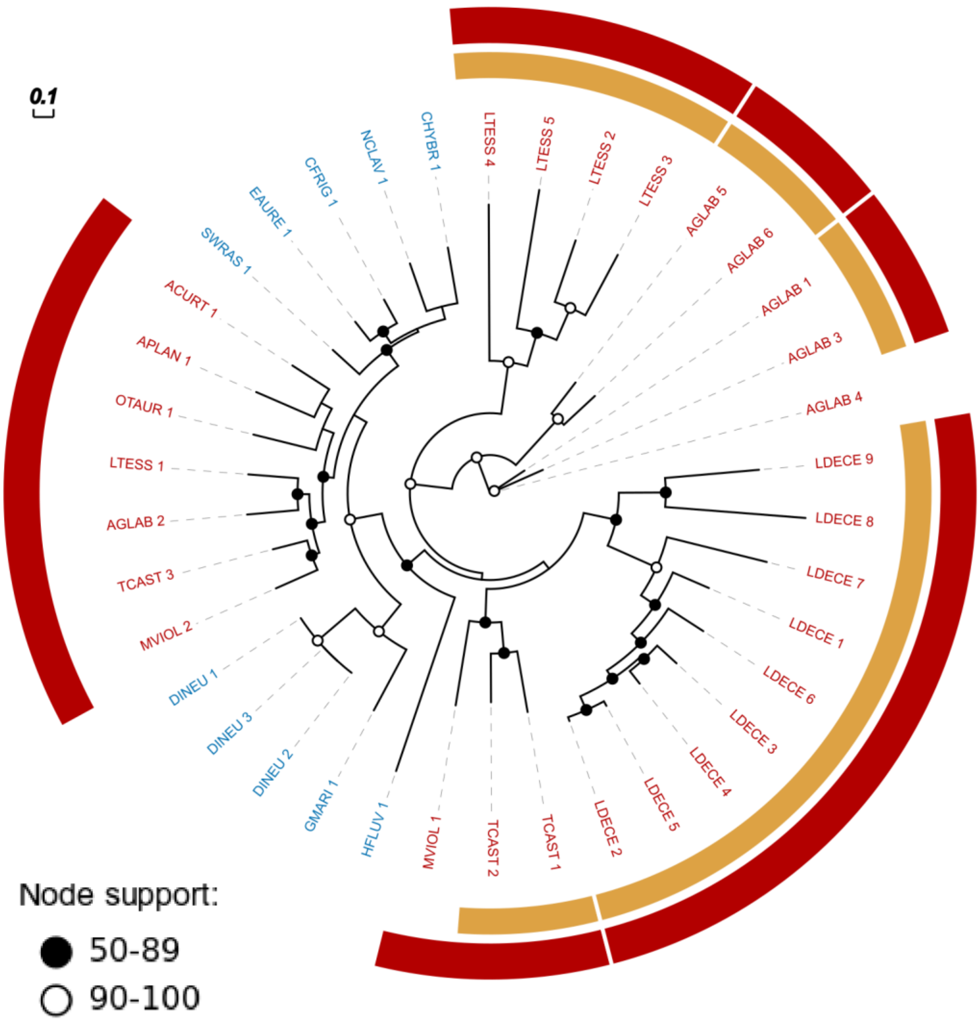

Supplementary Fig. S9. CYS_EOG8JDKNM

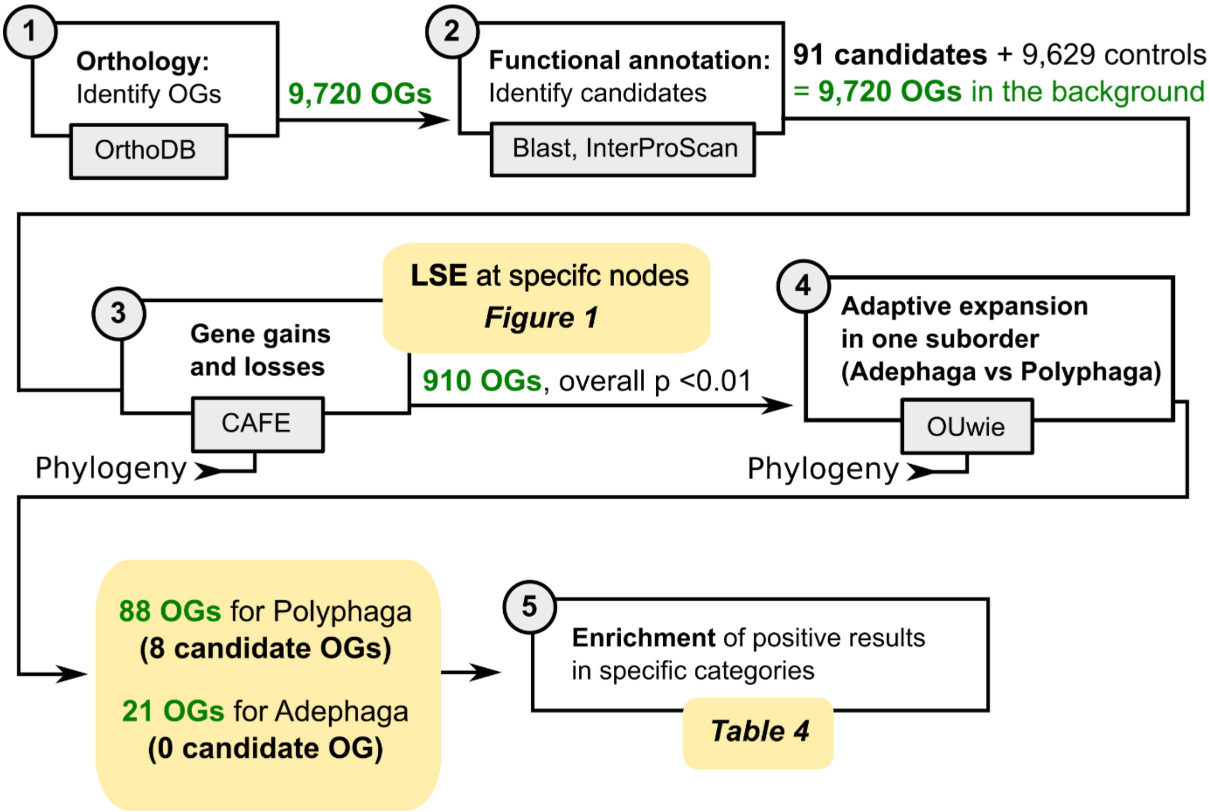

Supplementary Fig. S10. Chart summarizing the major steps leading to the main results (yellow areas). OGs=orthologous groups.

Supplementary References

Aronesty, E. (2011). ea-utils : “Command-line tools for processing biological sequencing data”;

Haas, B.J., Papanicolaou, A., Yassour, M., Grabherr, M., Blood, P.D., Bowden, J., Couger, M.B., Eccles, D., Li, B., Lieber, M., et al. (2013). De novo transcript sequence reconstruction from RNA-seq using the Trinity platform for reference generation and analysis. Nat. Protoc. *8*, 1494–1512.

Junier, T., and Zdobnov, E.M. (2010). The Newick utilities: high-throughput phylogenetic tree processing in the UNIX shell. Bioinforma. Oxf. Engl. *26*, 1669–1670.

Kent, W.J. (2002). BLAT--the BLAST-like alignment tool. Genome Res. *12*, 656–664.

McKenna D.D., Scully E.D., Pauchet Y., Hoover K., Kirsch R., Geib S.M., Mitchell R.F., Waterhouse R.M., Ahn S-J., Arsala D., et al., (2016). Genome of the Asian longhorned beetle (Anoplophora glabripennis), a globally significant invasive species, reveals key functional and evolutionary innovations at the beetle–plant interface. *Genome Biol* **17**: 227.

Misof, B., Liu, S., Meusemann, K., Peters, R.S., Donath, A., Mayer, C., Frandsen, P.B., Ware, J., Flouri, T., Beutel, R.G., et al. (2014). Phylogenomics resolves the timing and pattern of insect evolution. Science *346*, 763–767.

Peters, R.S., Krogmann, L., Mayer, C., Donath, A., Gunkel, S., Meusemann, K., Kozlov, A., Podsiadlowski, L., Petersen, M., Lanfear, R., et al. (2017). Evolutionary History of the Hymenoptera. Curr. Biol. *27*, 1013–1018.

Revell, L.J. (2012). phytools: an R package for phylogenetic comparative biology (and other things): phytools: R package. Methods Ecol. Evol. *3*, 217–223.

Seppey M., Pitteloud C., Emerson B.C., Alvarez N. (2018). Laparocerus tessellatus adult full-body transcriptome. https://doi.org/10.5281/zenodo.1336288

Vasilikopoulos A., Balke M., Beutel R.G., Donath A., Podsiadlowski L., Pflug J.M., Waterhouse R.M., Meusemann K., Peters R.S., Escalona H.E., et al. (2019). Phylogenomics of the superfamily Dytiscoidea (Coleoptera: Adephaga) with an evaluation of phylogenetic conflict and systematic error. Mol Phylogenet Evol.

Xie, Y., Wu, G., Tang, J., Luo, R., Patterson, J., Liu, S., Huang, W., He, G., Gu, S., Li, S., et al. (2014). SOAPdenovo-Trans: de novo transcriptome assembly with short RNA-Seq reads. Bioinformatics *30*, 1660–1666.
